# FoxO3 deficiency in cortical astrocytes leads to impaired lipid metabolism and aggravated amyloid pathology

**DOI:** 10.1111/acel.13432

**Published:** 2021-07-11

**Authors:** Shuqi Du, Feng Jin, Laure Maneix, Manasee Gedam, Yin Xu, Andre Catic, Meng C. Wang, Hui Zheng

**Affiliations:** ^1^ Huffington Center on Aging Baylor College of Medicine Houston TX USA; ^2^ Department of Molecular and Cellular Biology Baylor College of Medicine Houston TX USA; ^3^ Department of Pharmacology and Chemical Biology Baylor College of Medicine Houston TX USA; ^4^ Stem Cells and Regenerative Medicine Center Baylor College of Medicine Houston TX USA; ^5^ Graduate Program in Translational Biology and Molecular Medicine Baylor College of Medicine Houston TX USA; ^6^ Department of Molecular and Human Genetics Baylor College of Medicine Houston TX USA; ^7^ Howard Hughes Medical Institute Baylor College of Medicine Houston TX USA

**Keywords:** aging, Alzheimer's disease, astrocytes, FoxO3, mice, β‐amyloid

## Abstract

The rise of life expectancy of the human population is accompanied by the drastic increases of age‐associated diseases, in particular Alzheimer's disease (AD), and underscores the need to understand how aging influences AD development. The Forkhead box O transcription factor 3 (FoxO3) is known to mediate aging and longevity downstream of insulin/insulin‐like growth factor signaling across species. However, its function in the adult brain under physiological and pathological conditions is less understood. Here, we report a region and cell‐type‐specific regulation of FoxO3 in the central nervous system (CNS). We found that FoxO3 protein levels were reduced in the cortex, but not hippocampus, of aged mice. FoxO3 was responsive to insulin/AKT signaling in astrocytes, but not neurons. Using CNS *Foxo3*‐deficient mice, we reveal that loss of FoxO3 led to cortical astrogliosis and altered lipid metabolism. This is associated with impaired metabolic homoeostasis and β‐amyloid (Aβ) uptake in primary astrocyte cultures. These phenotypes can be reversed by expressing a constitutively active FOXO3 but not a FOXO3 mutant lacking the transactivation domain. Loss of FoxO3 in 5xFAD mice led to exacerbated Aβ pathology and synapse loss and altered local response of astrocytes and microglia in the vicinity of Aβ plaques. Astrocyte‐specific overexpression of FOXO3 displayed opposite effects, suggesting that FoxO3 functions cell autonomously to mediate astrocyte activity and also interacts with microglia to address Aβ pathology. Our studies support a protective role of astroglial FoxO3 against brain aging and AD.

## Graphical abstract

FoxO3 expression decreases with aging. CNS‐specific FoxO3 deficiency leads to aberrant cortical astrocyte activation. This is associated with metabolic defects in mitochondrial respiration, lipid consumption, and reduced Aβ uptake capacity. In an AD mouse model, loss of FoxO3 aggravates Aβ pathology while astrocytic FoxO3 overexpression significantly reduces the amyloid burden.

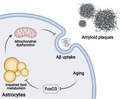

## 1 INTRODUCTION

Alzheimer's disease (AD), characterized by the deposition of β‐amyloid (Aβ) plaques and accumulation of neurofibrillary tangles, is the most common form of dementia occurring in aged populations. Although the etiology of AD remains elusive, aging is the greatest known risk factor. Among the various age‐associated conditions, strong evidence implicates that metabolic comorbidities, such as obesity and type 2 diabetes, increase the risk for late‐onset AD (Edwards Iii et al., 2019; Guerreiro & Bras, 2015). Key to the regulation of aging and metabolism are the Forkhead box O (FoxO) transcription factors, which act downstream of the insulin/insulin‐like growth factor signaling (IIS) pathway to mediate critical organismal processes, including growth control, reproduction, and lifespan regulation, and cellular responses such as energy utilization, metabolic homeostasis, autophagy, and stress resistance (Barthel et al., 2005; Brown & Webb, 2018; Gross et al., 2008; Martins et al., 2016). In both *Caenorhabditis elegans* and *Drosophila melanogaster*, reduction of IIS signaling leads to extended lifespan, and this effect requires FoxO/DAF16 and dFOXO, respectively (Friedman & Johnson, 1988; Friedman & Johnson, 1988; Kenyon et al., 1993; Woodling et al., 2020). This pathway is well‐conserved as FoxO3, the most prominent FoxO member in the mammalian system, is implicated in lifespan extension in caloric‐restricted mice (Shimokawa et al., 2015). In addition, single nucleotide polymorphisms of FoxO3 have been associated with extreme longevity in humans (Flachsbart et al., 2009; Willcox et al., 2008). Multiple cell types, from both peripheral tissues and central nervous system (CNS), have been shown to mediate the longevity‐promoting effect of FoxO3 through both autocrine and paracrine mechanisms (Bolukbasi et al., 2017; Demontis & Perrimon, 2010; Giannakou, et al. 2004; Hwangbo et al., 2004; Libina et al., 2003).

In the CNS, FoxO3 plays an essential role in maintaining the quiescent state of neural stem cells (NSCs) in the adult mouse brain. FoxO3 deletion drives NSC differentiation, resulting in the depletion of the NSC pool (Paik et al., 2009; Renault et al., 2009; Schäffner et al., 2018; Yeo et al., 2013). In addition, FoxO proteins have been shown to either promote neuronal survival or mediate apoptotic neuronal death, in dependence of external stimuli (Caballero‐Caballero et al., 2013; Lehtinen et al., 2006). Relevant to neurodegenerative diseases, FoxO3 has been shown to be protective against mutant Huntingtin (Voisin et al., 2020), α‐synuclein (Pino et al., 2014), and Aβ (Cohen et al., 2010). These effects were largely attributed to FoxO3 expression in neurons. However, little is known about the role of FoxO3 in other cell types of the brain.

Astrocytes are the most abundant cells in the CNS. Under physiological conditions, astrocytes mediate diverse biological activities including neural development, circuit function, neurotransmission, blood–brain barrier integrity, metabolic support, and synaptic regulation (Sofroniew & Vinters, 2010). During aging and under neuronal injury or neurodegenerative conditions such as AD, astrocytes become activated and display changes in morphology, gene expression, and function (Ben Haim et al., 2015; Sofroniew, 2020). Astrocytes produce and secrete various cytokines, chemokines, and growth factors (Cabezas et al., 2016; Choi et al., 2014; Glabinski et al., 1996; Lau & Yu, 2001; Strack et al., 2002). They also participate in the uptake of extracellular materials including Aβ peptides, facilitating the transport of these materials across the blood–brain barrier (Alarcon et al., 2005; Liu et al., 2017; Villarreal et al., 2014). Additionally, FoxO3 has been reported to control astrocyte proliferation through downregulation of cytokine‐induced activation of the cell cycle regulator p27^kip1^ (Cui et al., 2011). However, the cellular mechanisms mediating the FoxO3 function in astrocyte under physiological and AD relevant conditions remain to be explored.

Here, we discovered an age‐dependent reduction of FoxO3 expression in the mouse cortex, but not hippocampus. FoxO3 is highly sensitive to insulin signaling in astrocytes, but not in neurons. CNS‐specific *Foxo3* deficiency leads to aberrant cortical astrocyte activation. This is associated with metabolic defects in mitochondrial respiration, lipid consumption, and reduced Aβ uptake capacity. In an AD mouse model, loss of FoxO3 aggravates the Aβ pathology while astrocytic FoxO3 overexpression reduces the amyloid burden.

## 2 RESULTS

### 2.1 Age‐, region‐, and cell‐type‐specific regulation of FoxO3 expression and signaling

**FIGURE 1:**
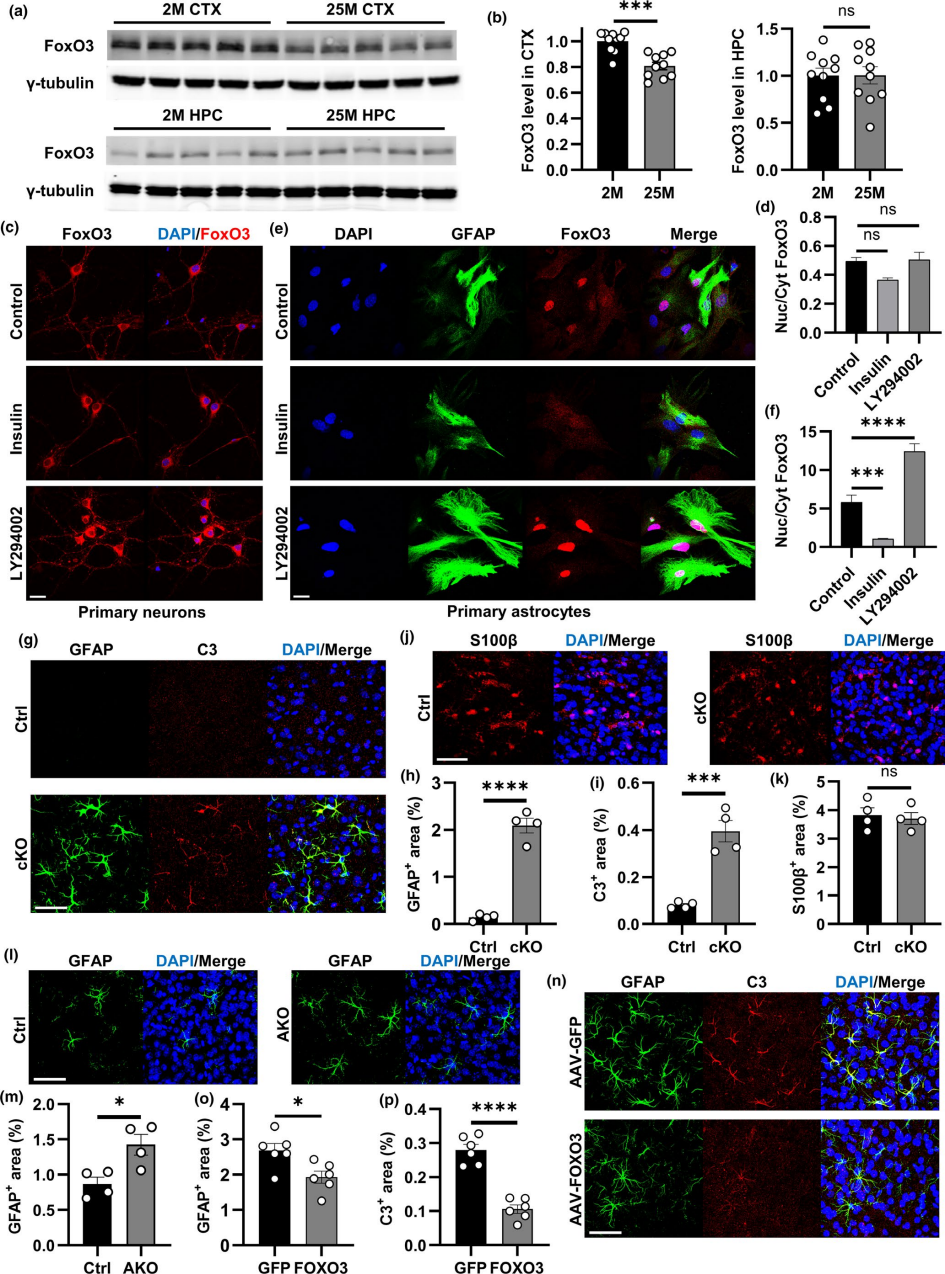
Regulation and function of FoxO3 in astrocytes. (a) Representative Western blots of FoxO3 protein levels in the cortex (upper panel) and hippocampus (lower panel) from mice at 2 months and 25 months. γ‐tubulin was used as the internal control. (b) Quantification of the levels of FoxO3 normalized to γ‐tubulin in (a). *N* = 10 (5 male +5 female)/group. (c) Representative confocal images showing FoxO3 subcellular localization in primary neurons via immunofluorescent staining after control (vehicle), insulin, or LY294002 treatment. Scale bar: 25 μm. (d) Quantification of nuclear/cytoplasmic FoxO3 ratio in (c). N_Control_=14; N_Insulin_ = 3; N_LY294002_=8. (e) Representative confocal images showing FoxO3 subcellular localization in primary astrocytes via immunofluorescent staining after control (vehicle), insulin, or LY294002 treatment. GFAP was used as a marker for astrocytes. Scale bar: 25 μm. (f) Quantification of nuclear/cytoplasmic FoxO3 ratio in (e). N_Control_=29; N_Insulin_ = 25; N_LY294002_=25. (g) Representative confocal images showing cortical astrocytes via immunofluorescent co‐staining of GFAP and C3 of cKO and Ctrl brain sections at 3 months of age. Scale bar: 50 μm. (h) Quantification of the GFAP‐positive percentage area in (g). *N* = 4/group. (i): Quantification of the C3‐positive percentage area in (g). *N *= 4/group. (j) Representative confocal images showing cortical astrocytes via immunofluorescent staining of S100β of cKO and Ctrl brain sections at 3 months of age. Scale bar: 50 μm. (k) Quantification of the S100β‐positive percentage area in (j). *N* = 4/group. (l) Representative confocal images showing cortical astrocytes via immunofluorescent staining of GFAP of AKO and Ctrl brain sections at 3 months of age. Scale bar: 50 μm. (m) Quantification of the GFAP‐positive percentage area in (l). *N* = 4/group. (n) Representative confocal images of C3 and GFAP co‐staining of cortical sections from 3‐month‐old *Foxo3* cKO mice with AAV‐FOXO3 or AAV‐GFP injections. Scale bar: 50 μm. O: Quantification of the GFAP‐positive percentage area in (n). *N* = 6/group. (p): Quantification of the C3‐positive percentage area in (n). *N* = 6/group. Male mice were used in both groups in (g–p). Data are presented as mean ±SEM. Significance determined by Student's t test or one‐way ANOVA with Tukey's multiple comparisons test. ns, not significant, *p < 0.05, ***p < 0.001, ****p < 0.0001

We first examined the expression of FoxO3 in the brain of young and old mice. Western blot analysis revealed significant reductions of FoxO3 in the cortex (CTX), but not hippocampus (HPC), of 25‐month‐old mice compared to 2‐month‐old samples (Figure 1a, b), indicating a possible impaired region‐specific FoxO function during aging. Next, we studied how FoxO3 responds to canonical signaling by examining its subcellular localizations under basal conditions and upon treatment with insulin, which sequesters FoxO in the cytoplasm through PI3K/AKT‐dependent phosphorylation, or treatment with the PI3K inhibitor LY294002, which promotes nuclear localization of FoxO. Immunofluorescent staining of N2a neuroblastoma (Figure S1a) and U87 astrocytoma (Figure S1c) cells showed that, under basal conditions, FoxO3 primarily resided in the cytoplasm, and this pattern was maintained upon insulin treatment. Treating the cells with LY294002 resulted in enhanced nuclear translocation in both cell types, as quantified by the nuclear‐to‐cytoplasmic ratios (Figure S1b and d). This enrichment was much more robust in U87 cells than in N2a cells, indicating higher insulin sensitivity in cells of astrocytic origin. Indeed, examination of FoxO3 localization in primary cultures of neurons revealed that FoxO3 remained in the cytoplasm under both insulin and LY294002 treatment conditions (Figure 1c, d). In contrast, in primary astrocyte cultures, FoxO3 was present in both nucleus and cytoplasm under basal conditions (Figure 1e), and insulin treatment decreased nuclear staining whereas LY294002 led to almost complete translocation to the nucleus (Figure 1e, f). To validate the effectiveness of the drugs, we performed Western blot analysis of total and phospho‐AKT (p‐AKT) in primary neuronal (Figure S1e, f) and astrocyte (Figure S1d, h) cultures treated with LY294002 or insulin. As expected, LY294002 led to diminished downstream phosphorylation of AKT while insulin treatment enhanced p‐AKT with no impact on total AKT levels in both cultures. Together, these results demonstrate that FoxO3 is highly sensitive to insulin signaling in cortical astrocytes.

### 2.2 FoxO3 inactivation in the brain induces cortical astrogliosis

To further investigate the FoxO3 function *in vivo*, we crossed the *Foxo3* floxed mice (Paik et al., 2007) with a Nestin‐Cre (Cre) line (Tronche et al., 1999) to create brain‐specific *Foxo3* conditional knockout (cKO) and used the littermate *Foxo3* ^fl/fl^ mice as controls (Ctrl). Since the Cre line alone has been reported to exhibit aberrant phenotypes (Harno et al., 2013), to assess a potential confounding effect of Cre to the cKO mice, we included the Cre, Ctrl, and cKO mice in our initial assessment. Quantitative PCR (qPCR) analysis documented comparable expression of *Foxo3* in Cre and Ctrl mice but a nearly complete elimination of *Foxo3* mRNA in cKO samples (Figure S2a). This is corroborated by Western blotting of FoxO3 proteins (Figure S2b). qPCR analysis of major cell type markers in the cortex and hippocampus showed no significant differences in the expression of *Syn*, a neuronal presynaptic marker, or *Aif1*, a marker for microglia, in Cre, Ctrl, and cKO samples (Figure S2c). In contrast, we recorded a significant upregulation of *Gfap*, a marker for reactive astrocytes, in the cortex, but not hippocampus, of *Foxo3* cKO mice, while no differences were detected between the Cre and Ctrl groups (Figure S2c). This was consistent with the immunofluorescence staining, which revealed low levels of cortical GFAP staining in Cre and Ctrl (Figure S2d, e), but a significant increase in GFAP immunoreactivity in cKO mice (Figure 1g), as quantified by the percentage area covered by GFAP‐positive staining (Figure 1h). These results argue against a possible off‐target effect of Nestin‐Cre and provide strong support that the aberrant cortical astrogliosis present in the cKO mice is caused by FoxO3 ablation.

To further characterize the astrogliosis phenotype, we performed co‐immunostaining with another reactive astrocyte marker Complement component 3 (C3) (Escartin et al., 2021; Liddelow et al., 2017), which revealed a more intense staining in the cortex of cKO mice (Figure 1g and i). In contrast, immunostaining for S100β, a general astrocyte marker (Cocchia, 1981; Ludwin et al., 1976), showed similar intensities between control and cKO mice (Figure 1j, k). Consistent with similar *Syn* and *Aif1* mRNA expression, the NeuN and Iba1 immunoreactivities were comparable between the Ctrl and cKO mice (Figure S2f–i), indicating no overt microglia or neuronal anomalies with *Foxo3* deficiency. Consistent with the increased *Gfap* mRNA levels only in cortex, but not in hippocampus, of cKO mice, similar GFAP and C3 staining was observed in the hippocampus of control and cKO samples (Figure S3a–c), as was S100β (Figure S3d, e). Likewise, hippocampal Iba1 immunoreactivities were also indistinguishable between the two groups (Figure S3f, g). Thus, loss of FoxO3 in CNS specifically induces cortical astrocytes to a reactive state without affecting hippocampal astrocytes or other brain cell types such as neurons and microglia.

The Nestin‐Cre is expressed in all neural lineages during embryonic development. To ascertain that the observed reactive astrogliosis in the cKO mice is due to the loss of astroglial FoxO3, but not neural stem cells, we generated astrocyte‐specific *Foxo3* knockout in adult brain (AKO) by crossing the *Foxo3* floxed allele with the Aldh1l1‐CreER line (Srinivasan et al., 2016). Treating the AKO and littermate controls with Tamoxifen at 8 weeks followed by analysis 4–5 weeks later observed mild but significant increases of GFAP immunoreactivity in the cortex of AKO mice compared to the Ctrl group (Figure 1l, m). We then prepared adeno‐associated virus (AAV) particles expressing either GFP or a FLAG‐tagged FOXO3 under the GFAP promoter. As we have shown previously (Chen et al., 2021; Martini‐Stoica et al., 2018), intracerebroventricular (i.c.v) injection of AAV‐GFP or AAV‐FOXO3 to the cKO mice at postnatal day 3 (P3) and analysis at 3 months documented efficient astrocyte expression (Figure S4a, b) and restoration of FoxO3 expression in AAV‐FOXO3‐injected mice (Figure S4c). GFAP and C3 immunostaining revealed a significant decrease in cortical astrogliosis in the cKO mice with AAV‐FOXO3 administration compared to AAV‐GFP‐injected controls (Figure 1n–p). This was corroborated by the downregulation of *Gfap* expression (Figure S4d). Iba1 immunointensities remained the same in both groups (Figure S4e, f). Thus, FoxO3 mediates cortical astrocyte activity through a cell autonomous mechanism.

### 2.3 FoxO3 deficiency alters astrocyte metabolism and function

**FIGURE 2:**
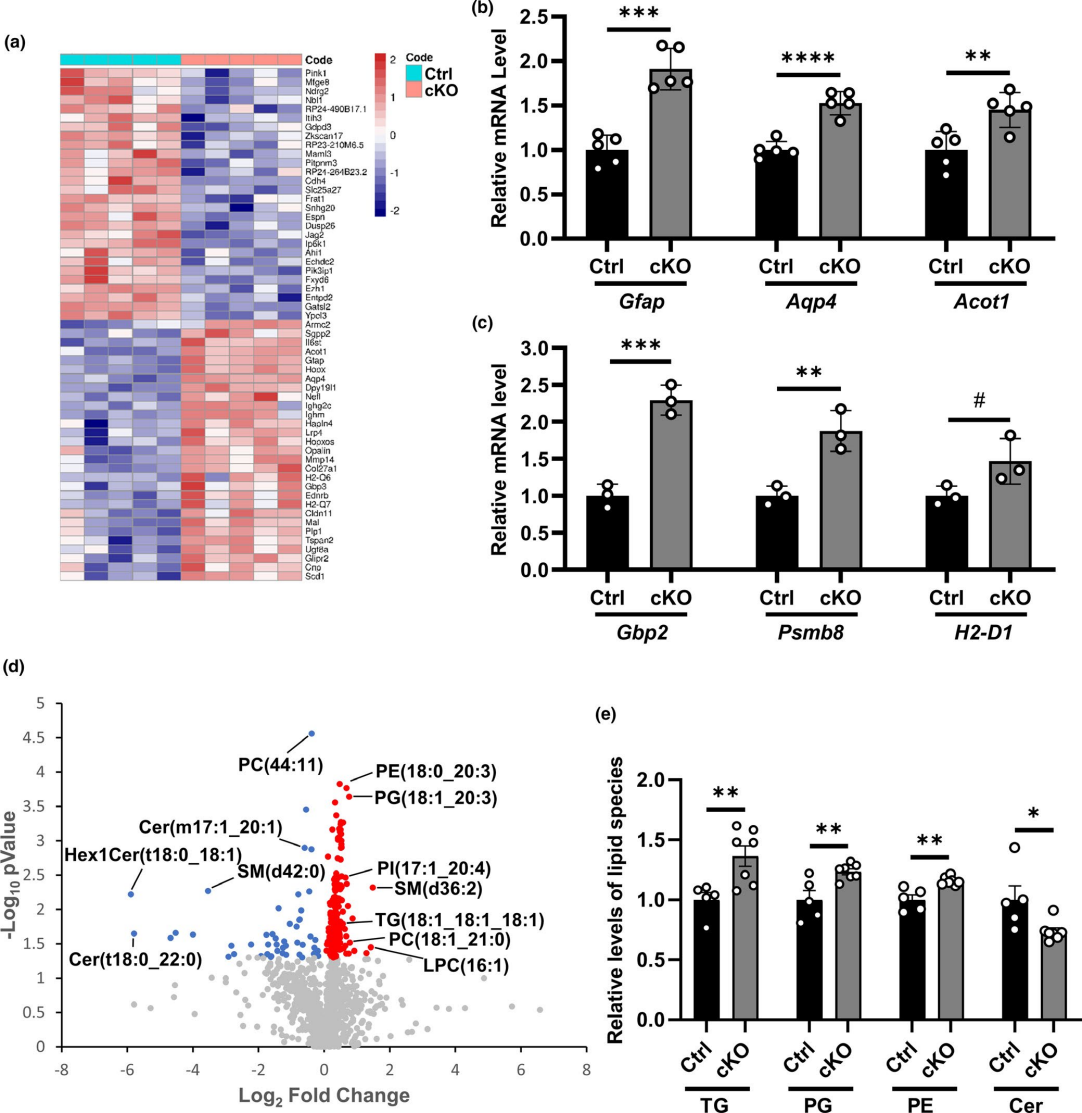
FoxO3 deletion alters the overall lipid profile in the brain. (a) Heatmap showing differentially expressed genes (DEGs) from the RNA‐seq analysis of cortex samples of *Foxo3* cKO and Ctrl mice at 3 months of age. FDR<0.05. *N* = 5. (b) qPCR analysis of the mRNA levels of *Gfap*, *Aqp4*, and *Acot1* in the cortex samples of *Foxo3* cKO and Ctrl mice at 3 months of age. *N* = 5. (c) qPCR analysis of the mRNA levels of *Gbp2*, *Psmb8*, and *H2*‐*D1* in the cortex samples of *Foxo3* cKO and Ctrl mice at 3 months of age. *N* = 3. (d) Volcano plot showing the fold change and *p* value distribution of the identified lipid species from lipidomics analysis of cortex samples of *Foxo3* cKO and Ctrl mice at 3.5 months of age. Significantly upregulated lipids (red), significantly downregulated lipids (blue), and lipids with insignificant change (gray) are labeled. N_Ctrl_=5; N_cKO_=7, *p *< 0.05. (e) Quantification of total abundance of triglyceride (TG), phosphatidylglycerol (PG), phosphatidylethanolamine (PE), and ceramide (Cer) among the differentially regulated lipids in (d). N_Ctrl_=5; N_cKO_=7. Male mice were used in both groups. Data are presented as mean ±SEM. Significance determined by Student's *t* test. #*p *< 0.1, *p < 0.05, **p < 0.01, ***p < 0.001, ****p < 0.0001

Given the region‐specific regulation of FoxO3, we performed RNA sequencing using cortex samples of *Foxo3* cKO mice and their control littermates to assess the impact of FoxO3 deficiency on gene expression. 58 differentially expressed genes (DEGs) were identified with a false discovery rate (FDR) less than 0.05 (Figure 2a). In agreement with the prominent astrogliosis phenotype in *Foxo3* cKO mice, we found significant increases in astroglial markers including *Gfap* and *Aqp4* in *Foxo3* cKO cortex, which were verified by qPCR analysis of bulk cortical samples (Figure 2b). Moreover, qPCR analysis revealed upregulation of *Gbp2*, *Psmb8*, and *H2*‐*D1*, markers of the A1 neurotoxic astrocyte in the cKO cortex (Liddelow et al., 2017; Zamanian et al., 2012) (Figure 2c). In addition, genes related to lipid metabolism known to be abundantly expressed in astrocytes, such as Acyl‐CoA Thioesterase 1 (*Acot1*, Figure 2b), Stearoyl‐CoA Desaturase 1 (*Scd1*), and Low‐Density Lipoprotein Receptor‐Related Protein 4 (*Lrp4*) (Kim et al., 2019; Polo‐Hernandez et al., 2014; Zhang et al., 2020), were also upregulated, indicating that FoxO3 deficiency may affect lipid homeostasis.

We thus performed untargeted lipidomic analysis using cortex samples of *Foxo3* cKO mice and their littermate controls. Partial least squares discriminant analysis (PLS‐DA) revealed clear separation between cKO and Ctrl groups (Figure S5a) with no change in the detected levels of the spiked internal standards (Figure S5b), indicating a shift in the overall lipid profile. We were able to identify 1302 distinct lipid species and generated a heatmap showing the abundance of the top 100 lipids in each sample with the most significant *p*‐values (Figure S5c). The volcano plot revealed 181 significantly upregulated and 49 significantly downregulated lipid species in the *Foxo3* cKO group (Figure 2d). We further classified these lipid molecules and calculated the total abundance of major lipid classes among them. We found increased levels of triglyceride (TG), phosphatidylglycerol (PG), and phosphatidylethanolamine (PE) while the abundance of ceramide (Cer) was significantly reduced in the cortex with FoxO3 deletion (Figure 2e). These results revealed a major role of FoxO3 in brain lipid regulation.

**FIGURE 3:**
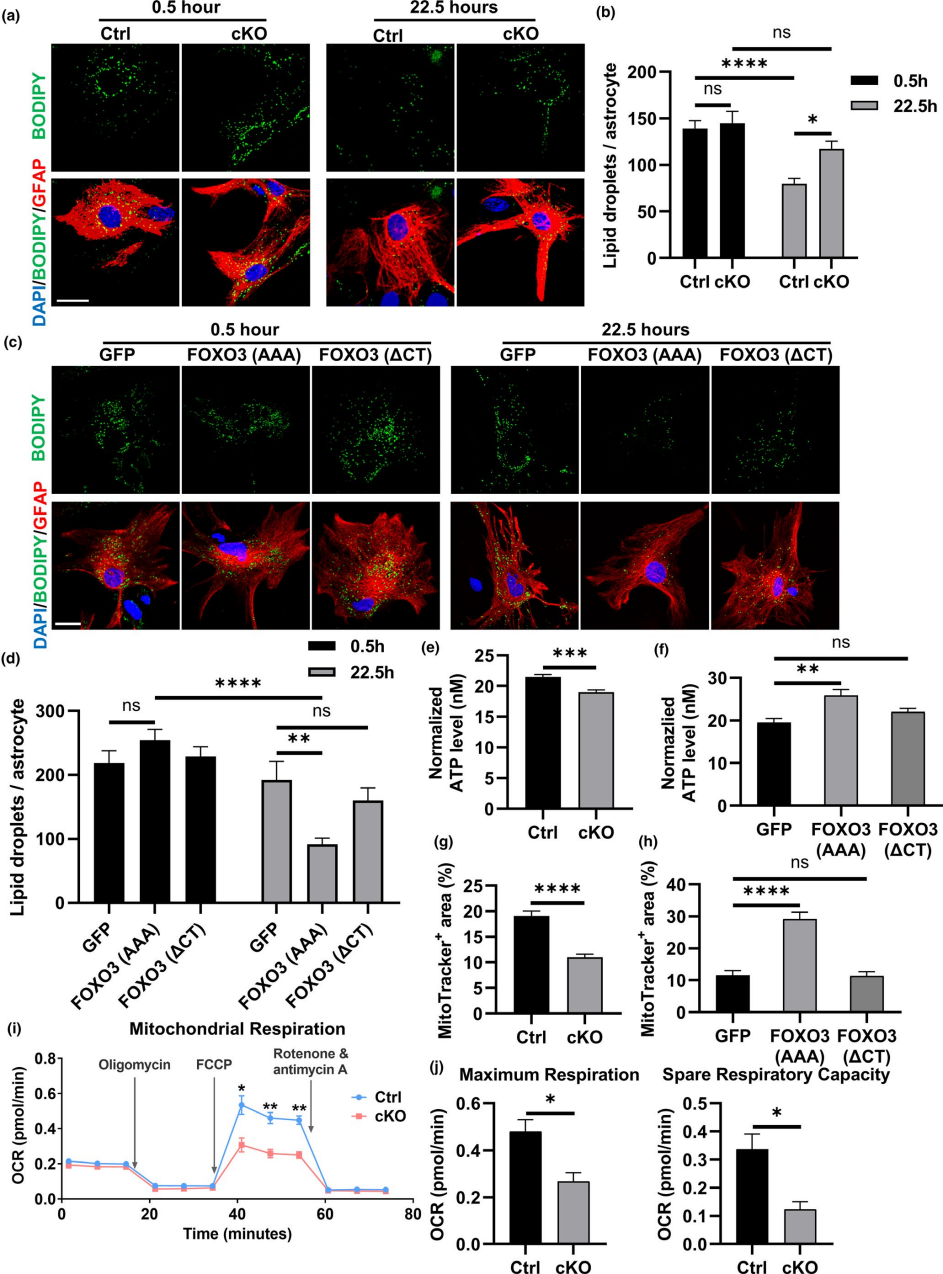
FoxO3 deficiency impairs lipid metabolism and mitochondrial function in cultured astrocytes. (a) Representative confocal fluorescent images of *Foxo3* cKO and Ctrl astrocytes after 8 h of 200 μM oleate‐BSA treatment followed by incubation in fresh medium for indicated times. Fixed cells were stained with BODIPY 493/503 (green) to label lipid droplets and immunostained with GFAP antibody (red) to label astrocytes. Scale bar: 25 μm. (b) Quantification of lipid droplets number in astrocytes in (a). *N* = 24. (c) Representative confocal fluorescent images of *Foxo3* cKO astrocytes infected with AAVs expressing GFP, FOXO3 (AAA) and FOXO3 (ΔCT) in the lipid consumption assay. Fixed cells were stained with BODIPY 558/568 (green) to label lipid droplets and immunostained with GFAP antibody (red) to label astrocytes. Scale bar: 25 μm. (d) Quantification of lipid droplets number in astrocytes in (c). *N* = 25. (e) ATP levels from a luciferase‐based ATP assay in *Foxo3* cKO and Ctrl astrocytes. Data were normalized to the protein levels determined by the BCA assay. *N* = 12. (f) ATP levels from a luciferase‐based ATP assay using *Foxo3* cKO astrocytes infected with AAVs expressing GFP, FOXO3 (AAA), and FOXO3 (ΔCT). Data were normalized to the protein levels determined by the BCA assay. *N* = 8. G: The percentage area of Mitotracker‐positive staining in *Foxo3* cKO and Ctrl astrocytes. N_Ctrl_=32; N_cKO_=37. (h) The percentage area of Mitotracker‐positive staining in *Foxo3* cKO astrocytes infected with AAVs expressing GFP, FOXO3 (AAA), and FOXO3 (ΔCT). *N* = 12. (i) Representative oxygen consumption rate (OCR) curve from Seahorse mito stress test in *Foxo3* cKO and Ctrl primary astrocytes. Oligomycin, FCCP and Rotenone & antimycin A were added in order to the culture medium. Results were normalized to the protein levels as determined by the BCA assay. *N* = 3 for each data point. (j) Quantification of the maximum respiration and spare respiratory capacity in (i). *N* = 3. Data are presented as mean ±SEM. Significance determined by Student's *t* test or one‐way ANOVA with Tukey's multiple comparisons test. ns, not significant, *p < 0.05, **p < 0.01, ***p < 0.001, ****p < 0.0001

Given the crucial role of astrocytes in lipid metabolism and the evidence that fatty acid oxidation primarily occurs in astrocytes (Barber & Raben, 2019; Ioannou et al., 2019), we next investigated whether FoxO3 deficiency in astrocytes impairs this function. We generated primary astrocyte cultures from *Foxo3* cKO mice and littermate controls. The astrocyte purity and the loss of FoxO3 in the cKO culture were validated by GFAP and FoxO3 immunostaining (Figure S6a). We first treated the cultures with oleate‐BSA for 8 h to load cells with excessive amount of extrinsic fatty acids and then measured the level of lipids at different time points after wash to determine the capacity of astrocytes in lipid catabolism. We found that cKO and Ctrl astrocytes had a comparable number of lipid droplets at 0.5 h. However, while there was a significant decrease in the number of lipid droplets in Ctrl astrocytes at 22.5 h, no significant reduction was observed in the cKO group (Figure 3a, b). To establish that the lipid phenotype is the direct result of FoxO3 loss, we infected the *Foxo3*‐deficient astrocytes with AAVs expressing GFP, a constitutively active FOXO3 variant (FOXO3 (AAA)) or an inactive FOXO3 variant with deletion of the transactivation domain (FOXO3 (ΔCT)) under the GFAP promoter. We found FOXO3 (AAA) but not FOXO3 (ΔCT) expression significantly reduced the internalized lipid droplets at the 22.5‐h time point compared to AAV‐GFP infected cells (Figure 3c, d). This result indicates that loss of FoxO3 in astrocytes leads to a diminished capacity to consume lipids while FOXO3 overexpression enhanced lipid turnover, and this activity requires canonical FOXO3 signaling.

It has been reported that lipid metabolism plays a fundamental role in maintaining the overall energy homeostasis in astrocytes (Lee et al., 2020). Indeed, measurement of total ATP using a luciferase‐based assay showed that *Foxo3* cKO astrocytes possessed a lower level of total ATP compared to the control culture (Figure 3e), which was reversed by FOXO3 (AAA) but not FOXO3 (ΔCT) expression (Figure 3f). We next assessed how the deficiency of FoxO3 impacts the metabolic function of mitochondria, a key organelle for cellular energy production. qPCR of extracted total DNA from astrocyte cultures revealed no changes in the relative levels of mitochondrial genes *Nd1* or *Rnr2* to the genomic gene *Hk2* between *Foxo3* cKO and control astrocytes (Figure S6b). MitoTracker Red CMXRos stains mitochondria and its intensity correlates with the mitochondrial membrane potential. We observed decreased MitoTracker staining in *Foxo3* null cells, suggesting impaired mitochondrial integrity and function (Figure 3g). Expression of FOXO3 (AAA), but not FOXO3 (ΔCT), significantly increased the area of positive MitoTracker staining (Figure 3h), suggesting a functional role of FoxO3 in maintaining mitochondrial homeostasis. Utilizing a Seahorse extracellular flux analyzer to quantify oxidative respiration, we found that cKO astrocytes had a significantly lower maximal stress respiration, measured by the oxygen consumption rates (OCR), than control cells after the injection of FCCP, a potent uncoupler of mitochondrial oxidative phosphorylation, while the OCR under other conditions were similar (Figure 3I). Quantification showed reduced mitochondrial maximum respiration and as a result, diminished respiratory reserve capacity in *Foxo3*‐deficient astrocytes (Figure 3J), indicating a weakened mitochondrial potential to enhance energy production under stress conditions. These results combined demonstrate impaired mitochondrial respiration in the absence of FoxO3 in astrocytes.

**FIGURE 4:**
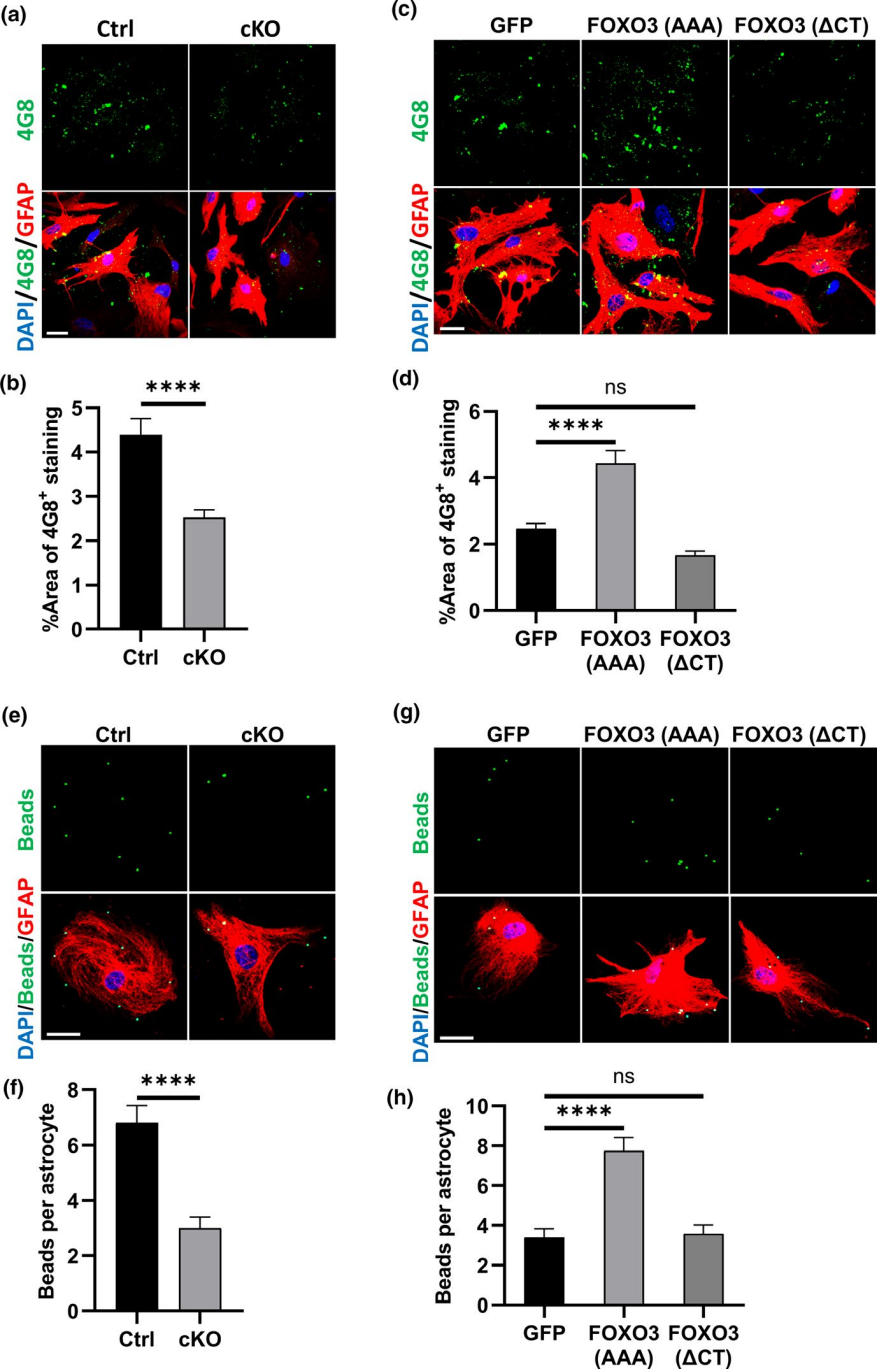
FoxO3 deficiency impairs Aβ uptake in astrocyte cultures. (a) Representative confocal fluorescent images of *Foxo3* cKO and Ctrl astrocytes after 24 h of 100 nM fibril Aβ treatment. Fixed cells were immnunostained with 4G8 antibody (green) and GFAP antibody (red) to label Aβ and astrocytes, respectively. Scale bar: 25 μm. (b) Quantification of percentage area of 4G8‐positive staining in astrocytes in (a). N_Ctrl_=38; N_cKO_=40. (c) Representative confocal fluorescent images of *Foxo3* cKO astrocytes infected with AAVs expressing GFP, FOXO3 (AAA) and FOXO3 (ΔCT) after Aβ treatment. Fixed cells were immnunostained with 4G8 antibody (green) and GFAP antibody (red) to label Aβ and astrocytes, respectively. Scale bar: 25 μm. (d) Quantification of percentage area of 4G8‐positive staining in astrocytes in (c). *N* = 20. (e) Representative confocal fluorescent images of bead (green) uptake in *Foxo3* cKO and Ctrl astrocytes. Fixed cells were immunostained with GFAP antibody (red) to label astrocytes. Scale bar: 50 μM. (f) Quantification of bead number in astrocytes in (e). *N* = 30. (g) Representative confocal fluorescent images of bead (green) uptake in *Foxo3* cKO astrocytes infected with AAVs expressing GFP, FOXO3 (AAA), and FOXO3 (ΔCT). Fixed cells were immunostained with GFAP antibody (red) to label astrocytes. Scale bar: 50 μM. (h) Quantification of bead number in astrocytes in (g). *N* = 25. Data are presented as mean ±SEM. Significance determined by Student's *t* test or one‐way ANOVA with Tukey's multiple comparisons test. ns, not significant, ****p < 0.0001

It has been shown that astrocytes could take up extracellular Aβ species to facilitate their clearance (Ries & Sastre, 2016). We reasoned that the altered metabolic properties in cKO astrocytes may affect their ability to take up extracellular Aβ. To test this possibility, we incubated fibrillar Aβ prepared from synthetic Aβ_1‐42_ peptides with astrocyte cultures for 24 h. After intensive wash, we fixed the culture and performed immunofluorescent staining with the anti‐GFAP and anti‐Aβ (4G8) antibodies to evaluate the level of internalized Aβ (Figure 4a). We found that there was significantly less intracellular Aβ in GFAP‐positive astrocytes in *Foxo3* cKO culture compared to controls (Figure 4b). Expression of FOXO3 (AAA) significantly restored the capacity of Aβ internalization in *Foxo3* cKO astrocytes while FOXO3 (ΔCT) had no effect (Figure 4c, d). To rule out the possibility that the decrease in internalized Aβ was caused by enhanced turnover, we measured the uptake of fluorescent latex beads which are degradation resistant. Consistent with the Aβ experiment, *Foxo3* null astrocytes internalized fewer beads than controls, validating a role of FoxO3 in extracellular uptake rather than intracellular degradation (Figure 4e, f). Consistently, the impaired beads uptake capacity in *Foxo3* null astrocytes could be enhanced by viral expression of FOXO3 (AAA), but not FOXO3 (ΔCT) (Figure 4g, h). Thus, astroglial FoxO3 deficiency leads to impaired metabolomic homeostasis and reduced phagocytic capacity, implicating a potential role of FoxO3 in Aβ pathology.

### 2.4 Astrocytic FoxO3 modulates glial properties and amyloid pathology in 5xFAD mice

We thus sought to determine whether FoxO3 deficiency could have a direct impact on Aβ pathology *in vivo*. We crossed *Foxo3* cKO mice with the 5xFAD mice, a mouse model with rapid progression of amyloid pathology (Oakley et al., 2006), to generate cKO; 5xFAD and their littermate 5xFAD mice. Western blot analysis showed no appreciable changes of full‐length APP (APP‐FL), APP C‐terminal fragments (APP‐CTFs), or the APP beta‐site cleaving enzyme BACE1 (Figure S7), suggesting that FoxO3 inactivation does not affect APP expression or processing.

**FIGURE 5:**
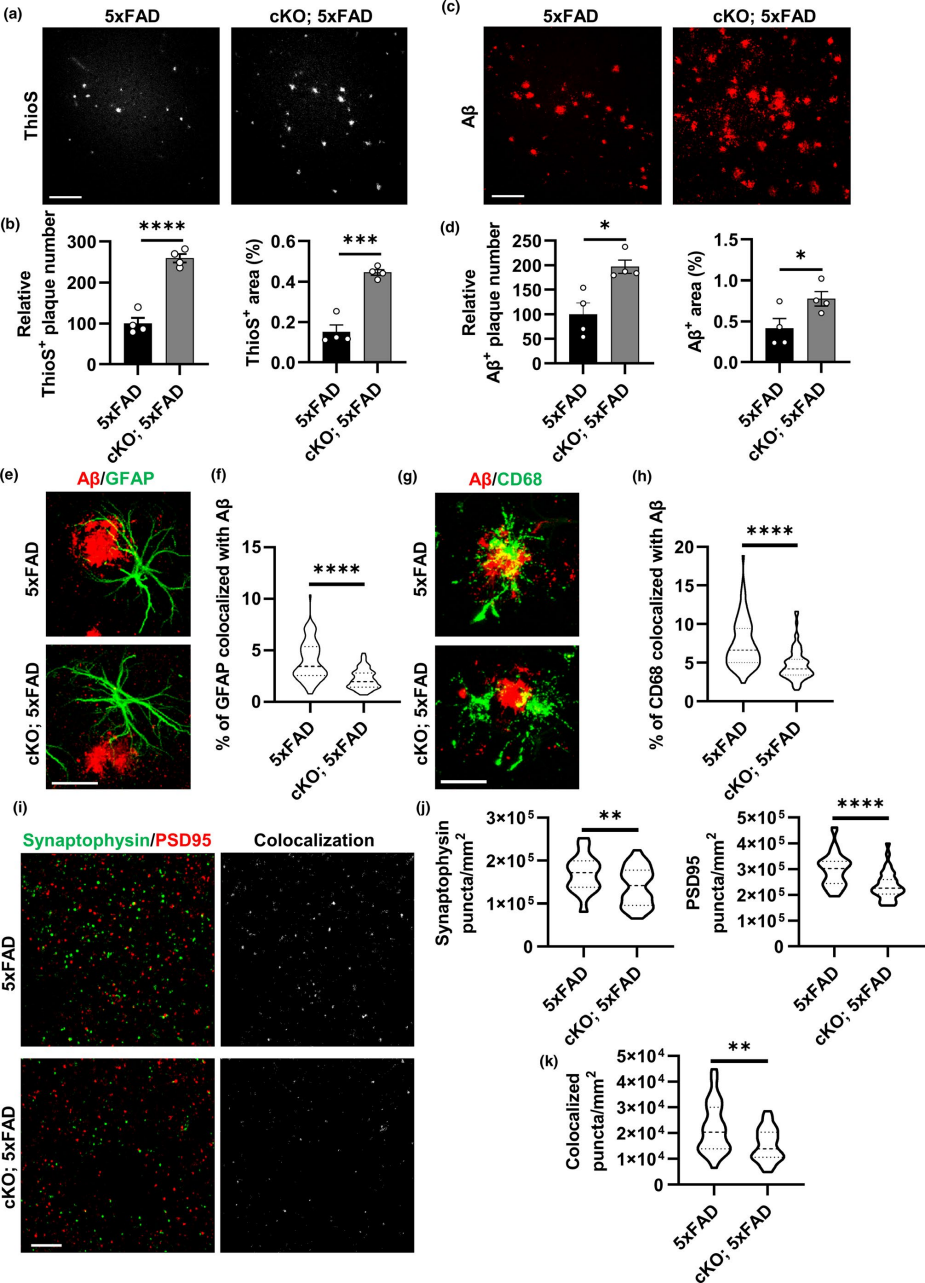
Loss of FoxO3 aggravates amyloid pathology and alters glial behaviors in 5xFAD mice. (a) Representative confocal images showing amyloid plaques in the cortex by Thioflavin S staining in 3.5‐month‐old *Foxo3* cKO; 5xFAD and 5xFAD mice. Scale bar: 100 μm. (b) Quantification of Thioflavin S‐positive plaque number and percentage area in the cortex. *N* = 4. (c) Representative confocal images showing amyloid plaques in the cortex by Aβ immunostaining in FoxO3 cKO; 5xFAD and 5xFAD mice. Scale bar: 100 μm. (d) Quantification of Aβ‐positive plaque number and percentage area in the cortex. *N* = 4. (e) Representative confocal images showing amyloid plaques and plaque‐associated reactive astrocytes via Aβ and GFAP co‐staining in *Foxo3* cKO; 5xFAD and 5xFAD mice. Scale bar: 30 μm. (f) Quantification of percentage of GFAP‐positive volume colocalized with Aβ in (e) using IMARIS software. *N* = 75. (g) Representative confocal images showing amyloid plaques and phagocytic microglia via Aβ and CD68 co‐staining in *Foxo3* cKO; 5xFAD and 5xFAD mice. Scale bar: 20 μm. (h) Quantification of percentage of CD68‐positive volume colocalized with Aβ in (g) using IMARIS software. *N* = 75. (i): Representative confocal images of immunofluorescent co‐staining of Synaptophysin and PSD95 in *Foxo3* cKO; 5xFAD and 5xFAD mice. Colocalized signals from two channels were also shown. Scale bar: 10 μm. (j) Quantification of the average number of Synaptophysin‐positive puncta and PSD95‐positive puncta per mm^2^ in (i). *N* = 36. (k) Quantification of the average number of Synaptophysin and PSD95 colocalized puncta number per mm^2^ in (i). *N* = 36. Male mice at 3.5 months of age were used in all analyses. Data are presented as mean ±SEM. Significance determined by Student's *t* test. *p < 0.05, **p < 0.01, ***p < 0.001, ****p < 0.0001

Thioflavin S (ThioS) staining of 5xFAD and cKO; 5xFAD male mouse brains at 3.5 months (Figure 5a, b) and 5 months (Figure S8a, b) documented increased dense‐core plaques in the absence of FoxO3 at both ages. This was also the case when female mice were analyzed at 3.5 months using methoxy‐X04 (X04) which also labels dense‐core plaques (Figure S8c, d). We chose male mice at 3.5 months for in‐depth analysis as the effect of FoxO3 ablation is most significant. In agreement with the ThioS staining, immunostaining with a pan anti‐Aβ antibody also revealed increased Aβ plaque pathology in the cortex of cKO; 5xFAD mouse brains compared to the 5xFAD mice (Figure 5c, d). In contrast, the Aβ pathology in the hippocampus was not significantly different in the presence or absence of FoxO3 (Figure S9a, b), further strengthening a cortex‐specific function of FoxO3 *in vivo*.

An advanced amyloid pathology is accompanied by a higher level of gliosis. We observed increased levels of GFAP‐positive astrocytes and Iba1‐positive microglia in cKO; 5xFAD mice compared to the 5xFAD mice (Figure S9c–e). Examination of astrocytes close to Aβ plaques by co‐staining of GFAP with Aβ (Figure 5e) or with X04 (Figure S9f), followed by confocal imaging and 3D reconstruction using the IMARIS software, revealed that the percentage of GFAP colocalized with Aβ (Figure 5f) and the density of astrocytes within the 50 μm radius of the X04‐marked plaque core (Figure S9g) were both reduced in the cKO; 5xFAD mice compared to the 5xFAD group. Unexpectedly, co‐immunofluorescence staining of Aβ with an anti‐CD68 antibody, a marker for phagocytic microglia, also showed a significantly reduced colocalization in the absence of FoxO3 (Figure 5g, h), indicating reduced microglia Aβ phagocytosis in cKO; 5xFAD mice. Co‐staining of X04 with Iba1 followed by 3D reconstruction documented lower microglia density per X04‐positive plaque in *Foxo3*‐deficient 5xFAD mice (Figure S9h, i). These results raise the intriguing possibility that FoxO3 deficiency not only leads to intrinsic changes in astrocytes but also impedes microglia morphology and its Aβ uptake capacity.

To determine the impact of FoxO3 on synaptic properties, we stained the brain sections with presynaptic marker Synaptophysin and post‐synaptic marker PSD95 and observed significant loss of both markers as well as their colocalized puncta in the cortex of cKO; 5xFAD mice compared to 5xFAD mice (Figure 5i–k). Quantification of NeuN‐positive neurons in the same area did not reveal significant changes (Figure S8j, k), indicating higher synaptic damage but no neuronal loss with FoxO3 deficiency.

**FIGURE 6:**
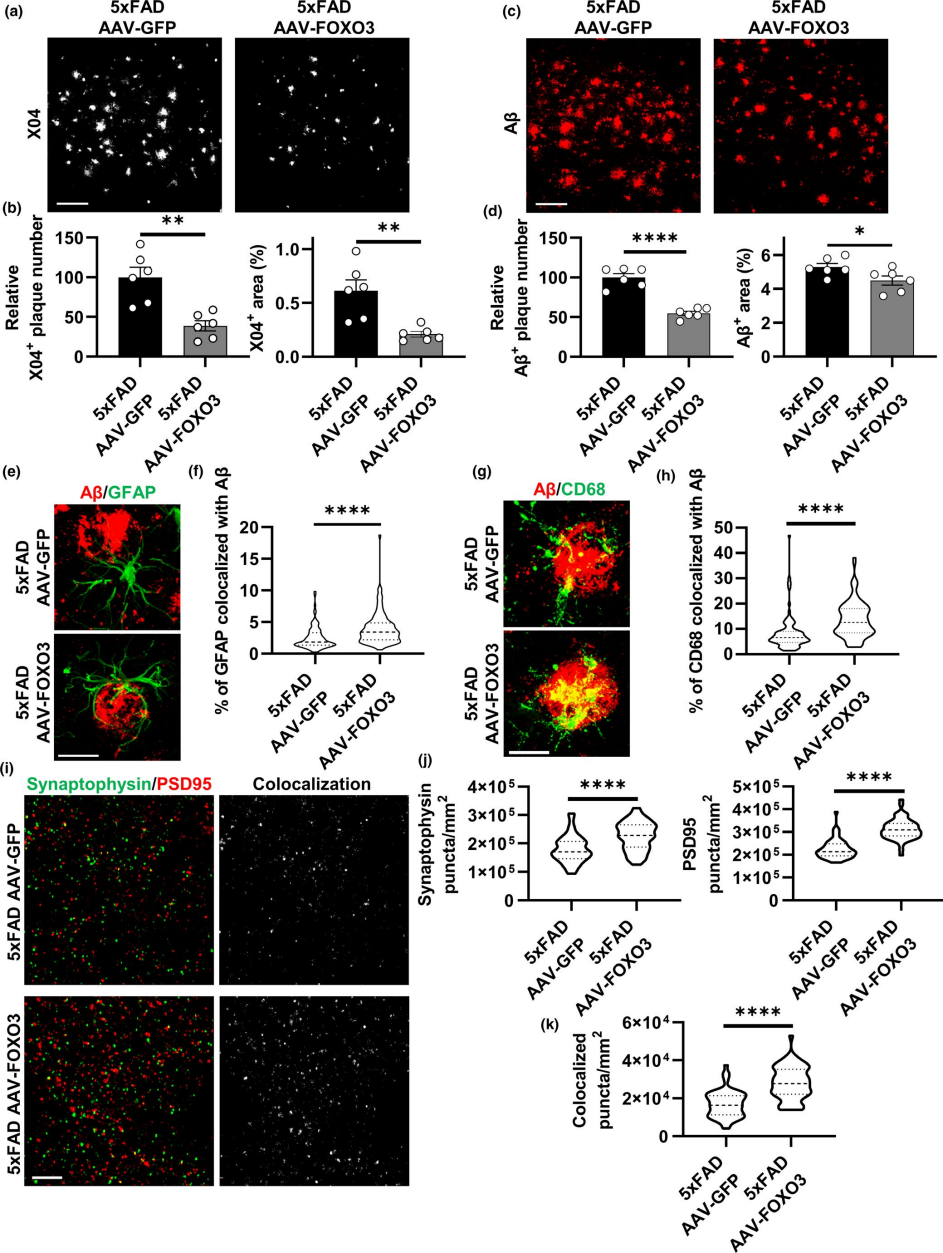
Astrocytic FoxO3 expression mitigates the pathological phenotypes in 5xFAD mice. (a) Representative confocal images showing amyloid plaques in the cortex by X04 staining of 5‐month‐old 5xFAD mice with AAV‐GFP or AAV‐FOXO3 injections. Scale bar: 100 μm. (b) Quantification of X04‐positive plaque number and percentage area in the cortex. *N* = 6. (c) Representative confocal images showing amyloid plaques in the cortex by Aβ staining of 5xFAD mice with AAV‐GFP or AAV‐FOXO3 injections. Scale bar: 100 μm. (d) Quantification of Aβ‐positive plaque number and percentage area in the cortex. *N* = 6. (e) Representative confocal images showing amyloid plaques and plaque‐associated reactive astrocytes via Aβ and GFAP co‐staining of 5xFAD mice with AAV‐GFP or AAV‐FOXO3 injections. Scale bar: 30 μm. (f) Quantification of percentage of GFAP‐positive volume colocalized with Aβ in (e) using IMARIS software. *N* = 90. (g) Representative confocal images showing amyloid plaques and phagocytic microglia via Aβ and CD68 co‐staining of 5xFAD mice with AAV‐GFP or AAV‐FOXO3 injections. Scale bar: 20 μm. (h) Quantification of percentage of CD68‐positive volume colocalized with Aβ in (g) using IMARIS software. *N* = 90. (i) Representative confocal images of immunofluorescent co‐staining of Synaptophysin and PSD95 of 5xFAD mice with AAV‐GFP or AAV‐FOXO3 injections. Colocalized signals from two channels were also shown. Scale bar: 10 μm. (j) Quantification of the average number of Synaptophysin‐positive puncta and PSD95‐positive puncta per mm^2^ in (i). *N* = 54. (k): Quantification of the average number of Synaptophysin and PSD95 colocalized puncta number per mm^2^ in (i). *N* = 54. Female mice at 5 months of age were used in all experiments. Data are presented as mean ±SEM. Significance determined by Student's *t* test. *p < 0.05, **p < 0.01, ****p < 0.0001

To provide additional evidence for an astroglial role of FOXO3, we transduced AAV‐GFAP‐FOXO3 or control AAV‐GFAP‐GFP to the P3 of 5xFAD mouse brains to test whether augmentation of astrocytic FOXO3 can reverse the Aβ and associated phenotypes. We collected the mice at 5 months of age as we expected an amelioration of Aβ pathology by FOXO3 expression. Staining of the brain sections of female 5xFAD mice with X04 (Figure 6a and S10a) or the pan‐Aβ antibody (Figure 6c and S10b) showed that, as expected, the AAV‐FOXO3‐injected group exhibited a much lower plaque burden compared to the AAV‐GFP group as quantified by X04‐positive or Aβ‐positive plaque area and number in the cortex (Figure 6b and d). Similar results were obtained when male mice were analyzed (Figure S10c and d). Co‐staining of GFAP with Aβ (Figure 6e) or X04 (Figure S10e) followed by 3D reconstruction revealed that the percentage of GFAP colocalized with Aβ (Figure 6f) and the density of astrocytes within the 50 μm radius of the plaque core (Figure S10f) were both increased in the AAV‐FOXO3‐injected group compared to AAV‐GFP‐injected controls. Likewise, Aβ and CD68 co‐staining showed a significantly higher colocalization of CD68 with Aβ in the AAV‐FOXO3 group (Figure 6g, h), consistent with the higher Iba1 density per X04‐positive plaque (Figure S10g, h). Similar to FoxO3 loss of function, astroglial FOXO3 expression did not alter neuronal density (Figure S10i, j), but it significantly increased levels of synaptic markers and their colocalized puncta in cortex compared to the control group with GFP expression (Figure 6i–k), in agreement with reduced plaque pathology. Taken together, these results suggest that astrocytic FoxO3 modulates amyloid pathology through both a cell autonomous effect and by influencing the recruitment and phagocytic function of plaque‐associated microglia.

## 3 DISCUSSION

Astrocytes are the major glial cells in the brain known to mediate metabolomic processes and energy homeostasis (Deitmer et al., 2019). However, how FoxO3 contributes to astrocyte function under physiological and pathological conditions is less understood. Here, we report a critical role of FoxO3 in mediating astrocyte function and AD neuropathology, and this activity is brain‐region and cell‐type dependent. Specifically, FoxO3 protein levels were reduced in the cortex, but not hippocampus of aged mice; FoxO3 was responsive to insulin/PI3K‐AKT signaling in astrocytes, but not neurons. Using CNS *Foxo3*‐deficient mice, we show that loss of FoxO3 led to cortical astrogliosis and lipid dysregulation *in vivo* and impaired lipid and mitochondrial metabolism and Aβ uptake in astrocyte cultures, the latter can be reversed by expressing a constitutively active form of FOXO3 but not a variant lacking the transactivation domain, indicating a canonical FOXO3 transcriptional activity in this regulation. Loss of CNS FoxO3 on 5xFAD background was associated with increased Aβ pathology and gliosis, impaired synaptic density, and altered local responses of astrocytes and microglia in the vicinity of Aβ plaques. Astroglial AAV‐FOXO3 overexpression led to the opposite effects. The loss‐ and gain‐of‐function studies combined support a model whereby FoxO3 functions cell autonomously to mediate astrocyte metabolic homeostasis and reactivity; it also interacts with microglia to facilitate its Aβ uptake and clearance. These activities combined likely contribute to the robust effects of FoxO3 on amyloid pathology.

The insulin/insulin‐like growth factor signaling (IIS) is an evolutionarily conserved pathway that couples cellular metabolism to nutrition availability. Despite its essential function for growth, sustained activity of this pathway in adulthood and during aging has been associated with reduced lifespan in diverse species (Alic & Partridge, 2011; Fontana et al., 2010). Extensive studies in *C*. *elegans*, *Drosophila*, and mammals have established a longevity‐promoting role of IIS blockade, and this effect can be conferred by targeting specific tissues or cell types (Bolukbasi et al., 2017; Demontis & Perrimon, 2010; Giannakou et al., 2004; Hwangbo et al., 2004; Libina et al., 2003). Relevant to the CNS, Taguchi *et al*. reported that reduced IIS via Nestin‐Cre deletion of insulin receptor substrate‐2 (*Irs2*) extends lifespan in mice (Taguchi et al., 2007). However, it remained unclear whether it is mediated by the loss of Irs2 in neurons or astrocytes or both. A recent study used genetic manipulations of the IIS pathway in different glial subtypes in the *Drosophila* brain and found that reduced insulin/PI3K signaling specifically in astrocyte‐like glia, but not in other glial subtypes, extends lifespan without delaying development and this effect is FoxO3 dependent (Woodling et al., 2020). Our result that the subcellular localization of FoxO3 in astrocytes is highly sensitive to insulin/PI3K signaling is in keeping with this idea and supports a conserved astrocytic insulin/PI3K/FoxO3 pathway in metabolism and lifespan regulation. Interestingly, neuronal FoxO3 seems to be less responsive to insulin signaling regulation. This could be explained by the existence of distinct FoxO3 regulatory pathways between the two cell types. It has been reported that, besides PI3K/AKT‐mediated phosphorylation, the subcellular localization of FoxO3 could also be affected by AKT‐independent phosphorylation (Greer et al., 2007; Huang et al., 2006; Yuan et al., 2008), by other post‐translational modifications such as acetylation (Beharry et al., 2014) and methylation (Yamagata et al., 2008), and through β‐catenin interaction (Essers et al., 2005). Thus, it is possible that these pathways may play more prominent roles in neuronal FoxO3 signaling. Indeed, neuronal FoxO3 has been implicated in apoptosis under stress conditions (Barthelemy et al., 2004; Gilley et al., 2003), while astrocytic FoxO3 apparently exerts protective function through metabolic regulation. Further investigations are required to better understand the cell‐type‐specific FoxO3 regulation and function in the brain.

Astrocyte heterogeneity is a well‐recognized feature in both physiological and disease conditions. Studies have demonstrated that astrocytes from different regions of the murine brain differ molecularly, morphologically, and functionally (Chai et al., 2017), and also display different sensitivities toward insults (Zhao & Flavin, 2000). We observed that *Foxo3* conditional knockout in the brain led to reactive astrogliosis marked by elevated GFAP and C3, but not S100β, immunoreactivities in the cortex, but not hippocampus where basal GFAP expression is high. Similarly, FoxO3 deficiency increased plaque burden in the cortical region of 5xFAD mice with minimum influence on hippocampal pathology. Intriguingly, the high level of GFAP in the hippocampus is accompanied by abundant C3 expression. The functional implication for this phenomenon remains to be established. It has been reported that astrocyte metabolic pathways are modulated by their activation status (Iglesias et al., 2017). It is thus possible that hippocampal astrocytes have a different metabolic profile than cortical astrocytes and, as a result, are less dependent on FoxO3. A region‐specific role of astrocytes has been demonstrated by Huang et al. (2020), which revealed that the transcription factor nuclear factor I‐A is required to maintain astrocyte function in the hippocampus, but not cortex, through region‐specific DNA binding. As a transcriptional factor, FoxO3 could mediate cortical‐specific functions through similar mechanisms. It has been proposed that astrocytes acquire distinct molecular phenotypes, being either neurotoxic (A1) or neuroprotective (A2), in response to different pathological conditions (Liddelow et al., 2017; Zamanian et al., 2012). Our qPCR analysis showed that *Foxo3* conditional knockout led to upregulated mRNA levels of a small subset of A1 fingerprints, including *Gbp2*, *Psmb8*, and *H2*‐*D1* in mouse cortex, suggesting that *Foxo3*‐deficient astrocytes may share some A1‐like features. However, increasing evidence indicates that this binary polarization of reactive astrocytes fails to capture their phenotypic diversity (Escartin et al., 2021). Therefore, it is likely that reactive astrocytes induced by FoxO3 depletion are more nuanced but remain to be clearly defined.

Lipid homeostasis plays a critical role in brain physiology, and its aberrant accumulation has been implicated in pathological processes such as AD (Bales, 2010; Ledesma et al., 2012; Sultana et al., 2013). Unlike neurons that do not prefer fatty acids as an energy source, astrocytes are active in consuming, producing, storing, and releasing lipid species, through which they support neuronal activities (Barber & Raben, 2019; Ioannou et al., 2019; Schonfeld & Reiser, 2013). FoxO3 has been reported to regulate lipid metabolism in peripheral tissues (Tao et al., 2013; Tao et al., 2013; Wang et al., 2019). We found that brain‐specific FoxO3 deletion not only altered the expression of genes related to lipid metabolism but also changed the abundance of many lipid molecules. Interestingly, results from our RNA sequencing and lipidomic analysis both revealed lipid pathway dysregulation in FoxO3 null brains. For example, increased expression of *Acot1* and *Scd1* can both enzymatically elevate the cellular level of oleic acid (C18:1), and, indeed, our lipidomic analysis was able to capture increased abundance of TG (18:1_18:1_18:1) (presumably oleic acid triglyceride) in the *Foxo3* cKO brain. These results are also in agreement with our *in vitro* lipid consumption assay, in which we found *Foxo3* cKO astrocytes have impaired capacity to consume extrinsic fatty acids. Excess free fatty acids in the cytoplasm are reported to be toxic to the cells and may disrupt mitochondrial membrane integrity (Nguyen et al., 2017). Consistently, we observed decreased MitoTracker staining, indicating a reduction in mitochondrial membrane potential. Notably, *Acot1* gene, which were found upregulated in the cKO cortex, encodes an enzyme that competes substrates (Acyl‐CoA) with mitochondrial fatty acid β‐oxidation (Hunt et al., 2006). Thus, the deregulation of *Acot1* upon *Foxo3* deficiency could initiate a molecular pathway that leads to impaired fatty acid catabolism and subsequent mitochondrial failure in astrocytes. Of particular relevance, it has been reported that mitochondrial dysfunction caused a reactive astrogliosis in the murine cortex under physiological conditions (Fiebig et al., 2019), which was in keeping with the *Foxo3* cKO phenotype.

The reduced spare respiratory capacity in *Foxo3* cKO astrocytes suggests a state where cells were no longer able to boost their respiration to secure extra supply to resolve pathological conditions. In the case of Aβ pathology, it is possible that *Foxo3*‐deficient astrocytes have limited capacity to clear accumulating Aβ in the extracellular matrix as shown by the reduced Aβ uptake *in vitro* and decreased colocalization between Aβ and GFAP in *vivo*. The fact that heightened Aβ pathology could be reversed by astroglial expression of FOXO3 supports a cell autonomous mechanism of FoxO3 in astrocytes. Interestingly, we observed that changes of astroglial FoxO3 are also accompanied by altered microglial behaviors in response to plaque pathology. Since Nestin‐Cre is not expressed in microglia, the microglial phenotypes are likely non‐cell autonomous. It is known that astrocytes actively communicate with other cells, including microglia, through physical interactions and via the release of glial transmitters and secreted molecules and organelles (Sofroniew & Vinters, 2010). Astroglial FoxO3 inactivation may influence microglia morphology and function through one or more of these mechanisms that remain to be defined. The cell autonomous and non‐cell autonomous effects of FoxO3 could synergistically affect amyloid pathology in 5xFAD mice.

Overall, while we attempted to provide mechanistic understanding of the *in vivo* phenotypes using *in vitro* systems, our studies are limited by the inherent differences between the two systems and the challenges of validating the cell culture studies in mouse models. Nevertheless, our work revealed an important role of FoxO3 in regulating astrocyte metabolism *in vitro* and lipid homeostasis and amyloid pathology *in vivo*, and this effect is cell‐type and brain‐region specific. Combined with the reduced expression of FoxO3 in the aging brain, our results support the concept that elevating astrocytic FoxO3 may reverse cortical astrogliosis and associated functional impairment in aging and Alzheimer's disease.

## 4 EXPERIMENTAL PROCEDURES

### 4.1 Mice, AAV injections, and analysis

*Foxo3^fl^*^/^*^fl^* (Paik et al., 2007), Nestin‐Cre (Tronche et al., 1999), Aldh1l1‐CreER (Srinivasan et al., 2016), and 5xFAD (Oakley et al., 2006) mouse lines were obtained from the Jackson Laboratories. The aged C57BL/6J mice were obtained from the aging rodent colony of the National Institute on Aging. The sample size was determined based on previous studies (Lian et al., 2016). Both male and female mice were used, and these are specified in the results and figure legends. Investigators were blinded to the group identities during data collection and analysis. For AAV injections, postnatal day 3 pups were anesthetized via hypothermia and injected i.c.v. free‐hand with 2.5 × 10^10^ viral particles per side using a 28‐gauge needle attached to a Hamilton syringe as described previously (Chen et al., 2021; Martini‐Stoica et al., 2018). RNA sequencing of total RNA was performed using the Illumina platform. Untargeted lipidomics was carried using a Vanquish UPLC and a Lumos orbitrap mass spectrometer (Thermo Fisher Scientific). The detailed methods for these experiments and other standard procedures including qPCR, Western blotting, immunofluorescence staining and associated image acquisition and analysis are provided in Supplementary Information.

### 4.2 Cell culture, treatment, and analysis

The preparation of primary astrocyte and neuronal cultures, AAV infections, and lipid consumption, ATP measurement, Seahorse mito stress test, and Aβ and beads uptake assays are described in detail in Supplementary Information. All *in vitro* assays were performed at least three times with a minimum of 3 technical replicates per experiment.

### 4.3 Quantification and statistical analysis

All data were analyzed with GraphPad Prism v.7.04 and presented as mean ±SEM (*p < 0.05, **p < 0.01, ***p < 0.001 and ****p < 0.0001). For simple comparisons, Student's t test was used. For multiple comparisons, one‐way ANOVA followed by Tukey's multiple comparisons test was utilized and is specified for each experiment in the figure legends. All samples or animals were included in the statistical analysis unless otherwise specified.

## CONFLICT OF INTEREST

None declared.

## AUTHOR CONTRIBUTIONS

SD and HZ designed the study. SD performed all experiments and data analysis except the lipodomics which was performed by FJ with guidance from MCW. LM and AC provided support for the Seahorse assays, MG and YX offered technical support for FACS and mouse brain analyses, respectively. SD wrote and HZ edited the paper. All authors provided input, read and approved the manuscript.

## Supporting information

Supplementary Material

## Data Availability

All data associated with this study are present in the manuscript or as supplementary information. Submissions of RNA sequencing and lipodomics data to NCBI are in process.
