# Supplementary material for: FoxO3 deficiency in cortical astrocytes leads to impaired lipid metabolism and aggravated amyloid pathology
Source: Aging Cell. 2021 Jul 11;20(8):e13432. doi: 10.1111/acel.13432 (PMC8373366; doi:10.1111/acel.13432)
Supplement: Supplementary file 1 — Supplementary Material [file ACEL-20-e13432-s001.pdf]

Supplementary Information

Experimental Procedures

Reagents

APP (Abcam, ab32136), CD68 (BIORAD, MCA1957GA), FoxO3 (CST, 2497S), Aβ (CST, 2454S), 4G8 (Biolegend, 800701), γ-tubulin (Sigma, T6557), GFAP (Millipore, MAB360), Iba1 (Wako, 019-19741), BACE-1 (EMD Millipore, AB5832), S100β (Sigma, S2644), C3 (Hycult Biotech, HM1045), NeuN (Millipore, MAB377), p-AKT (CST, 9271S), AKT (CST, 4691S), Synaptophysin (R&D system, AF5555), PSD95 (Invitrogen, 51-6900), DAPI (Invitrogen, D1306), LY294002 (CST, 9901S), Insulin (Novus Biologicals, NBP199193), Oleic acid (Sigma,O1383), BODIPY 493/503 (Invitrogen, D3922), BODIPY 588/568 (Invitrogen, D3835), Latex beads (Sigma, L1030), Human beta Amyloid (1-42) Recombinant Protein (Invitrogen, 03-111), Thioflavin S (Sigma-Aldrich, T1892), Methoxy-X04 (Tocris, 4920), Pierce BCA Protein Assay Kit (Thermo Fisher Scientific, 23227), Passive Lysis Buffer (Promega, E1941), ATP Determination Kit (Invitrogen, A22066), Seahorse XFp FluxPak (Agilent, 103022-100), Seahorse Cell Mito Stress Test Kit (Agilent, 103010-100), HRP-conjugated secondary antibodies (EMD Millipore, AP308P, AP307P), AlexaFlour-conjugated secondary antibodies (Thermo Fisher Scientific, A21202, A21206, A31571, A31572, A31573, A31574), ECL (Thermo Scientific, 32106, 34096), protease and phosphatase inhibitor cocktails (Roche, 04693116001, 04906845001), IRDye secondary antibodies (LI-COR, 926-68073, 926-32212)

Plasmid construction

Human FOXO3 coding sequence containing a N-terminal FLAG tag or its mutants were cloned into pAAV-GFAP-GFP by replacing the EGFP sequence. pAAV-GFAP-GFP, pAAV-GFAP-FOXO3, pAAV-GFAP-FOXO3 (AAA) and pAAV-GFAP-FOXO3 (ΔCT) vectors were used to generate AAV 2/8 by the Gene Vector Core (Baylor College of Medicine) as described previously [1]. AAVs were dialyzed against phosphate buffered saline without Mg^{2+} and Ca^{2+}, and the titer was determined by real time PCR. pAAV-GFAP-GFP was a gift from Bryan Roth (Addgene plasmid # 50473). pECE-FOXO3, pECE-FOXO3 (AAA) and pECE-FOXO3 (ΔCT) were gifts from Michael Greenberg (Addgene plasmid # 8630, #1788, #1796) [2-4].

Cell culture

N2a cells were purchased from ATCC. U87 cells were a kind gift from Dr. Benjamin Deneen at Baylor College of Medicine. Culture of N2a and U87 cells were maintained in DMEM with 10% fetal bovine serum and 1% v/v Pen/Strep, and passaged 3 times a week.

For insulin and LY294002 treatment, N2a or U87 cells were seeded on sterile glass coverslips in a 24-well plate at 50,000 cells/cm^2. At 40% confluency, vehicle, 100 nM insulin and 50 µM LY294002 were added to the culture medium for one hour, followed by wash in ice-cold PBS, fixation, immunostaining and imaging. Experiment was performed twice with each in triplicate.

Primary neurons and primary astrocytes were prepared using a previously described protocol [5]. In brief, cortices were isolated from new-born pups (P0 for primary neurons and P3 for primary astrocytes) in ice-cold dissection medium (HBSS + 10 mM HEPES + 0.6 g/L glucose + 1% Pen/Strep), with meninges removed. The tissue was then finely minced and digested in 0.125% trypsin at 37°C for 15 minutes followed by the addition of trypsin inhibitor (40 µg/mL) and DNase (250 µg/mL). The tissue was then centrifuged at 1000 x g for 5 minutes, resuspended in neuron culture media (Neurobasal medium supplemented with 2% B27, 0.5 mM L-glutamine, 0.4% v/v Pen/Strep) or astrocyte culture media (DMEM + 10% fetal bovine serum + 1% Pen/Strep), and triturated. The cell suspension was centrifuged and resuspended one more time to remove tissue debris. For neuronal culture, cells were plated onto poly-D-lysine (PDL)-coated glass coverslips at 50,000 cells/cm^2 and incubated at 37°C with 5% CO2 for 2 weeks before experiments. For astrocyte culture, cells were plated on PDL-coated T75 flasks at 50,000 cells/cm^2. The mixed glial culture was allowed to reach confluency prior to shaking at 220 rpm overnight at 37°C to remove unwanted cell types (microglia, oligodendrocytes, neurons, and fibroblasts). After a 24-hour recovery in fresh culture medium, astrocytes were trypsinized in 0.25% trypsin/EDTA and replated onto appropriate PDL-coated coverslips for experiments. Media was changed every 3-4 days.

For AAV infections in the primary culture, *Foxo3* cKO astrocytes were split into 3 T-25 flasks at 7x10^5 cells per flask. After 4 hours to allow cells to attach, AAV-GFAP-GFP, AAV-GFAP-FOXO3 (AAA), AAV-GFAP-FOXO3 (ΔCT) were added to each flask at MOI 10^5, respectively. After 24-hour incubation, AAV-containing medium was replaced with fresh culture medium. Expand the culture to T-75 flask when they are confluent. 6 days after AAV infection, astrocytes were trypsinized and replated into 96 well plates or 24 well plates with coverslips for subsequent treatments.

Lipid consumption assay

20 mM sodium oleate solution was prepared by adding 100 µL oleic acid to 15.9 mL of 12.6 mM NaOH solution and incubating the mix at 70 °C for 30 minutes with multiple times of inversions. Aliquots of 50 µL of 1M NaOH were added to the fatty acid solution followed by 5 minutes

incubation at 70 °C until micelles are no longer visible. 3.5 mL of the 20 mM sodium oleate solution was added dropwise to pre-warmed 11.6 mL of the 5% BSA solution in PBS to make the oleate/BSA complexes. The mix was then added to 335 mL of the cell culture medium and sterilized by passing through a 0.22 μm bottle-top filter. The final concentration of oleate in the medium was 200 μM. *Foxo3* cKO and Ctrl astrocytes were seeded on PDL-coated coverslips in 24-well plates at 50,000 cells per well. At 60% confluency, cells were treated with 200 μM oleate-BSA for 8 hours, followed by 3 times of wash with ice-cold PBS and incubation in fresh warm culture medium for 0.5 or 22.5 hours. Astrocytes were then fix and stained with BODIPY to label lipid droplets and immunostained with GFAP antibody to label astrocytes. Experiment was performed three times with each in 4 replicates.

ATP assay

Foxo3 cKO and Ctrl astrocytes were seeded in 96-well plates at 20,000 cells per well. At 90% confluency, cells were washed with ice-cold PBS and lysed in 1 × passive lysis buffer, followed by pipetting for 10 times. The lysates were split equally into two aliquots. One aliquot was transferred to a white opaque 96-well plate for the measurement of the ATP concentration using an ATP determination kit. The other aliquot was transferred to a clear 96-well plate for the measurement of the protein concentration using the BCA assay. The final ATP level was calculated by normalizing the ATP concentration in a well to its corresponding protein concentration. Experiment was performed three times with each in 8-12 replicates.

MitoTracker staining

Foxo3 cKO and Ctrl astrocytes were seeded on PDL-coated coverslips in 24-well plates at 50,000 cells per well. At 60% confluency, MitoTracker Red CMXRos was added into culture medium at 100 nM and incubated for 30 minutes. Cells were washed with PBS, counterstained with DAPI, and then mounted and imaged with confocal microscopy (Leica TCS SPE). The percentage area of MitoTracker Red positive staining in cytoplasm was quantified with the Image J software (NIH). Experiment was performed three times with each in 4 replicates.

Seahorse mito stress test

Seahorse mito stress tests were performed on Agilent Seahorse XFp Analyzer. Seahorse sensor cartridges were incubated in calibrant solution (Agilent, 100840-000) overnight. On the day of assay, *Foxo3* cKO and Ctrl astrocytes were seeded on Cell Tak (Corning)-coated Seahorse microplates at 20,000 cells per well in the assay medium (DMEM + 1mM sodium pyruvate + 2

mM glutamine + 10 mM glucose). Cell were then centrifuged at 200 g for 1 minute with no break to allow attachment to the plate. The OCR was measured in real-time during the assay, following the sequential injection of oligomycin (2 μM), FCCP (0.5 μM), and rotenone / antimycin A (0.5 μM). After analysis, the cells in the wells were lysed, and the protein concentrations were determined by BCA assay and used for normalization. Experiment was performed six times with each in triplicate.

Aβ uptake assay

Aβ fibrils were prepared from the synthetic $A\beta_{1-42}$ peptides following the protocol described previously [6]. In brief, lyophilized peptides were dissolved in hexafluoroisopropanol (HFIP) and incubated at room temperature for 2 hours. The HFIP was evaporated in a fume hood, allowing the formation of Aβ films. The film was then resuspended in DMSO, sonicated in a water bath for 10 minutes, diluted in 10 mM HCl to 100 μM, and incubated at 37°C for 24 hours.

Foxo3 cKO and Ctrl astrocytes were seeded on PDL-coated coverslips in 24-well plates at 50,000 cells per well. At 60% confluency, Aβ fibrils were added to the cell culture medium at a working concentration of 100 nM and incubated for 24 hours. Cells were washed intensively with PBS for 4 times before they were fixed and immunostained with 4G8 and an anti-GFAP antibody. Experiment was performed twice with each in 4 replicates.

Beads uptake assay

Aqueous green-fluorescent latex beads of 1 μm diameter were preopsonized in FBS for 1 hour at 37°C before dilution in cell culture medium. The final concentrations for beads and FBS in medium were 0.05% (v/v) and 0.25% (v/v), respectively. *Foxo3* cKO and Ctrl astrocytes were seeded on PDL-coated coverslips in 24-well plates at 50,000 cells per well. At 60% confluency, culture medium was replaced with bead-containing medium and the culture was incubated at 37°C for 1 hour. Cells were washed with PBS and fixed following by immunostaining of GFAP. Experiment was performed twice with each in 4 replicates.

RNA extraction, reverse transcription, and qPCR

Total RNA was extracted from mouse forebrain, cortex, or hippocampus samples with TRIzol reagent (Invitrogen), and was subjected to DNase digestion to remove the contaminating genomic DNA. Reverse transcription was performed using iScript Reverse Transcription Supermix (BIO-RAD). Quantitative PCR was done using SYBR Green Master Mix (RIO-RAD) on a BIO-RAD CFX384 Touch Real-Time PCR Detection System. The reaction mixes (10 μL) included 5 μL 2x

SYBR Green Master Mix, 0.1 μL each of sense and antisense primer at 50 μM, 4.8 μL of cDNA (diluted to 50 ng/μL). Primers were designed on NCBI Primer-BLAST. *18S rRNA*, *Gapdh* and *Pgk1* were used as internal controls for the analysis. The relative levels of expression were quantified and analyzed by using Bio-Rad CFX manager. The real-time value for each sample was averaged and compared using the comparative ΔΔCT method.

**Gene**	**Forward (5'-3')**	**Reverse (3'-5')**
Foxo3	CAAACGGCTCACTTTGTCCC	ATGGAGTTCTTCCAGCCCGC
Syn	ACTACTCCTCCTCGGCTGAA	GGAAGTCCATCATTGGCCCTT
Gfap	AGAAAGGTTGAATCGCTGGA	CGGCGATAGTCGTTA
Aif1	CAGACTGCCAGCCTAAGACA	AGGAATTGCTTGTTGATCCC
Aqp4	TCAGCATCGCTAAGTCCGTC	GGTTTCCATGAACCGTGGTG
Acot1	GCCACCCCGAGGTAAAAGG	CATCCTTGAGGCCATCCTTG
Gbp2	GGGGTCACTGTCTGACCACT	GGGAAACCTGGGATGAGATT
Psmb8	CAGTCCTGAAGAGGCCTACG	CACTTTCACCCAACCGTCTT
H2-D1	TCCGAGATTGTAAAGCGTGAAGA	ACAGGGCAGTGCAGGGATAG
Nd1	CTAGCAGAAACAAACCGGGC	CCGGCTGCGTATTCTACGTT
Rnr2	CCGCAAGGGAAAGATGAAAGAC	TCGTTTGGTTTCGGGGTTTC
Hk2	GCCAGCCTCTCCTGATTTTAGTGT	GGGAACACAAAAGACCTCTTCTGG
18s	CCATTCGAACGTCTGCCCTAT	GTCACCCGTGGTCACCATG
Gapdh	CACCATCTTCCAGGAGCGAG	CCTTCTCCATGGTGGTGAAGAC
Pgk1	GACATCTCCTAGTTTGGACAGTG	CAACGAGCGGTTCCGATG

Western blotting

Mouse forebrain, cortex or hippocampus samples were lysed in RIPA buffer (TBS with 1% NP-40 (Thermo Fisher Scientific), 1% sodium deoxycholic acid (EMD Millipore), 0.1% sodium dodecyl sulfate (EMD Millipore), and protease and phosphatase inhibitor cocktails (Roche)). Lysates were sonicated and centrifuged at 20,000 × g for 15 min to spin down the debris. Supernatants were boiled with loading buffer, used for SDS-PAGE and transferred to PVDF membranes. After incubation with primary and secondary antibodies, the signals were detected with film using ECL (Pierce) or Odyssey image system (LI-COR). The signal intensity was quantified using ImageJ (NIH). The biological replicates are shown in the western blot figures.

Histology and immunofluorescence

Cells grown on coverslips were washed in ice-cold PBS after the removal of culture medium, followed by fixation with 4% paraformaldehyde at room temperature for 20 min. After fixation, coverslips were gently washed with PBS. Coverslips were then incubated overnight in primary antibody in blocking solution (TBS + 0.4% Triton X-100 + 2% donkey serum) at 4°C. Coverslips were then washed in PBS followed by incubation with secondary antibodies for 2 hours in blocking buffer at room temperature. Coverslips were then stained with DAPI at room temperature for 20 min and washed with PSB. Finally, coverslips were mounted with mounting medium and imaged by confocal microscopy (Leica TCS SPE).

Mouse brains were collected after saline perfusion and fixed overnight with 4% paraformaldehyde in PBS. Then the brains were dehydrated in 30% sucrose in TBS and sectioned on a sliding microtome in 30 μm increments. The sections were washed twice in TBS and incubated overnight with primary antibodies in blocking solution (TBS + 0.4% Triton X-100 + 2% donkey serum) at 4°C. After washing, the sections were incubated for 2 hours at room temperature with secondary antibodies conjugated with AlexaFluor (Invitrogen). Then, sections were stained with DAPI for 20 min and washed extensively in TBS for at least 3 times. Floating sections were mounted on glass slides and allowed to dry prior to adding mounting medium. Images were taken on a confocal microscope (Leica SPE) or EVOS Cell Imaging System (Thermo Fisher Scientific). The Leica TCS SPE is equipped with 405, 488, 561, and 635-nm laser lines and HC PL APO 10X/0.40, HC PL APO 20X/0.70 CS, ACS APO 40X/1.15 OIL CS, ACS APO 63X/1.30 OIL CS objectives.

For Thioflavin S staining, brain sections were incubated with 0.002% Thioflavin S in TBS for 8 minutes, followed by rinsing twice in 50% ethanol for 1 minute. Then brain sections were washed in TBS for 10 min for 3 times before mounting and imaging. For methoxy-X04 staining, antibody-stained brain sections were dried onto slides overnight, rehydrated in PBS for 30 seconds, placed in 40% EtOH/PBS for 30 seconds, dyed with 1 μM methoxy-X04 in 40% EtOH/PBS for 30 seconds, rinsed in 40% EtOH/PBS for 30 seconds, then in 70% EtOH/PBS for 30 seconds, in 90% EtOH/PBS for 30 seconds and dip twice in xylene, then allowed to dry once again before mounting and imaging.

Image quantification

After immunofluorescence staining, confocal images were captured and the percentage area of positive fluorescent staining and number of immunoreactive cells were quantified using the Image J software (NIH). For quantification of percentage area and number of Thioflavin S, X04 or Aβ

stained amyloid plaques, sections were scanned using an EVOS FL auto system. Images were then analyzed by ImageJ and the whole cortex or hippocampus area was quantified.

RNA-sequencing

Total RNA from cortices of 3-month-old *Foxo3* cKO mice and their littermate controls was extracted using TRIzol and purified on the RNeasy Mini column (Qiagen) with DNase digestion. RNAseq analysis was performed by Novogene using Illumina NovaSeq platforms with paired-end 150 bp sequencing strategy. Raw reads were first aligned to the *Mus musculus* genome (UCSC mm10) using TopHat v2.0.9 with default parameters (-r 100 –p 8) [7]. The gene model was obtained from https://ccb.jhu.edu/software/tophat/igenomes.shtml. Then, htseq-count function of HTSeq was used to accumulate the number of aligned reads that fall under the exons of the gene (union of all the exons of the gene) to present the expression of each gene. Differential gene expression analysis was carried out using the DESeq2 package in the R environment [8]. Differentially expressed genes (DEGs) were identified with the false-discovery rate (FDR) of 0.05%.

Lipidomics assay

Brain cortices from *Foxo3* cKO and Ctrl mice were dissected in ice-cold PBS, frozen and crushed into powder in liquid nitrogen. 20 mg of each sample was collected in 1.5 mL tubes and homogenized in 200 µL of 50 mM $(NH_4)HCO_3$ solution. Samples were normalized based on protein concentrations from BCA assay. 10 µL of splash lipidomix Mass Spec Standard (Avanti, 330707) was spiked in each sample before the extraction of lipids. Lipids were then extracted using methanol, chloroform and water following a previously reported method [9, 10]. The extracted samples were dried in a vacufuge and resuspended in 200 µL isopropanol and methanol (50:50, vol/vol). The samples were analyzed using a Vanquish UPLC and a Lumos orbitrap mass spectrometer (Thermo Fisher Scientific). The mobile phase A was 5 mM ammonium formate with 0.1% formic acid in water and acetonitrile (50:50, vol/vol) and mobile phase B consists of 2-propanol, acetonitrile, and water (88:10:2, vol/vol). A reverse phase column-Thermo Accucore Vanquish C18+ was used to separate the lipids which were detected in both positive and negative electrospray ionization modes. Mass spectra were acquired in full-scan and data dependent MS2 modes. The settings used for ionization were sheath gas flow rate 50, auxiliary gas flow rate 10, sweep gas flow rate 1 mL/min, positive spray voltage 3500 Volt and negative spray voltage 2500 Volt. The resolution for MS1 is 120k. The mass scan range is 250-1200; maximum injection time was 50 ms; AGC target was 200,000; RF lens was 45%. For MS2 scanning, 20 dependent scans

were acquired in each cycle. The MS2 resolution was 30k; HCD was used to fragment precursor ions with stepped collision energy 25, 30, 35; AGC target was 50000. High-throughput analysis of lipidomic data were performed using Lipidsearch software (Thermo Fisher Scientific) [11, 12]. Lipid quantification was based on precursor ion area. MS2 data were used for identification by matching product ion spectra to Lipidsearch library. Both precursor and product ion mass tolerance were set at 5 ppm. M-score threshold was set 2.0. Then both the positive and negative data were aligned based on retention time tolerance 0.1 min and exact mass of lipid. The final data were filtered based on preferred ion adduct for each lipid class. The statistical analysis was done by MetaboAnalyst 5.0 [13].

Supplementary References

1. Martini-Stoica, H., et al., *TFEB enhances astroglial uptake of extracellular tau species and reduces tau spreading.* J Exp Med, 2018. **215**(9): p. 2355-2377.
2. Brunet, A., et al., *Stress-dependent regulation of FOXO transcription factors by the SIRT1 deacetylase.* Science, 2004. **303**(5666): p. 2011-5.
3. Brunet, A., et al., *Akt promotes cell survival by phosphorylating and inhibiting a Forkhead transcription factor.* Cell, 1999. **96**(6): p. 857-68.
4. Tran, H., et al., *DNA repair pathway stimulated by the forkhead transcription factor FOXO3a through the Gadd45 protein.* Science, 2002. **296**(5567): p. 530-4.
5. Lian, H., et al., *IkappaBalpha deficiency in brain leads to elevated basal neuroinflammation and attenuated response following traumatic brain injury: implications for functional recovery.* Mol Neurodegener, 2012. **7**: p. 47.
6. Stine, W.B., Jr., et al., *In vitro characterization of conditions for amyloid-beta peptide oligomerization and fibrillogenesis.* J Biol Chem, 2003. **278**(13): p. 11612-22.
7. Kim, D., et al., *TopHat2: accurate alignment of transcriptomes in the presence of insertions, deletions and gene fusions.* Genome Biol, 2013. **14**(4): p. R36.
8. Love, M.I., W. Huber, and S. Anders, *Moderated estimation of fold change and dispersion for RNA-seq data with DESeq2.* Genome Biol, 2014. **15**(12): p. 550.
9. Vantaku, V., et al., *Expression of ganglioside GD2, reprogram the lipid metabolism and EMT phenotype in bladder cancer.* Oncotarget, 2017. **8**(56): p. 95620-95631.
10. Piyarathna, D.W.B., et al., *Distinct Lipidomic Landscapes Associated with Clinical Stages of Urothelial Cancer of the Bladder.* Eur Urol Focus, 2018. **4**(6): p. 907-915.
11. Yamada, T., et al., *Development of a lipid profiling system using reverse-phase liquid chromatography coupled to high-resolution mass spectrometry with rapid polarity switching and an automated lipid identification software.* J Chromatogr A, 2013. **1292**: p. 211-8.
12. Taguchi, R. and M. Ishikawa, *Precise and global identification of phospholipid molecular species by an Orbitrap mass spectrometer and automated search engine Lipid Search.* J Chromatogr A, 2010. **1217**(25): p. 4229-39.
13. Chong, J., et al., *Using MicrobiomeAnalyst for comprehensive statistical, functional, and meta-analysis of microbiome data.* Nat Protoc, 2020. **15**(3): p. 799-821.

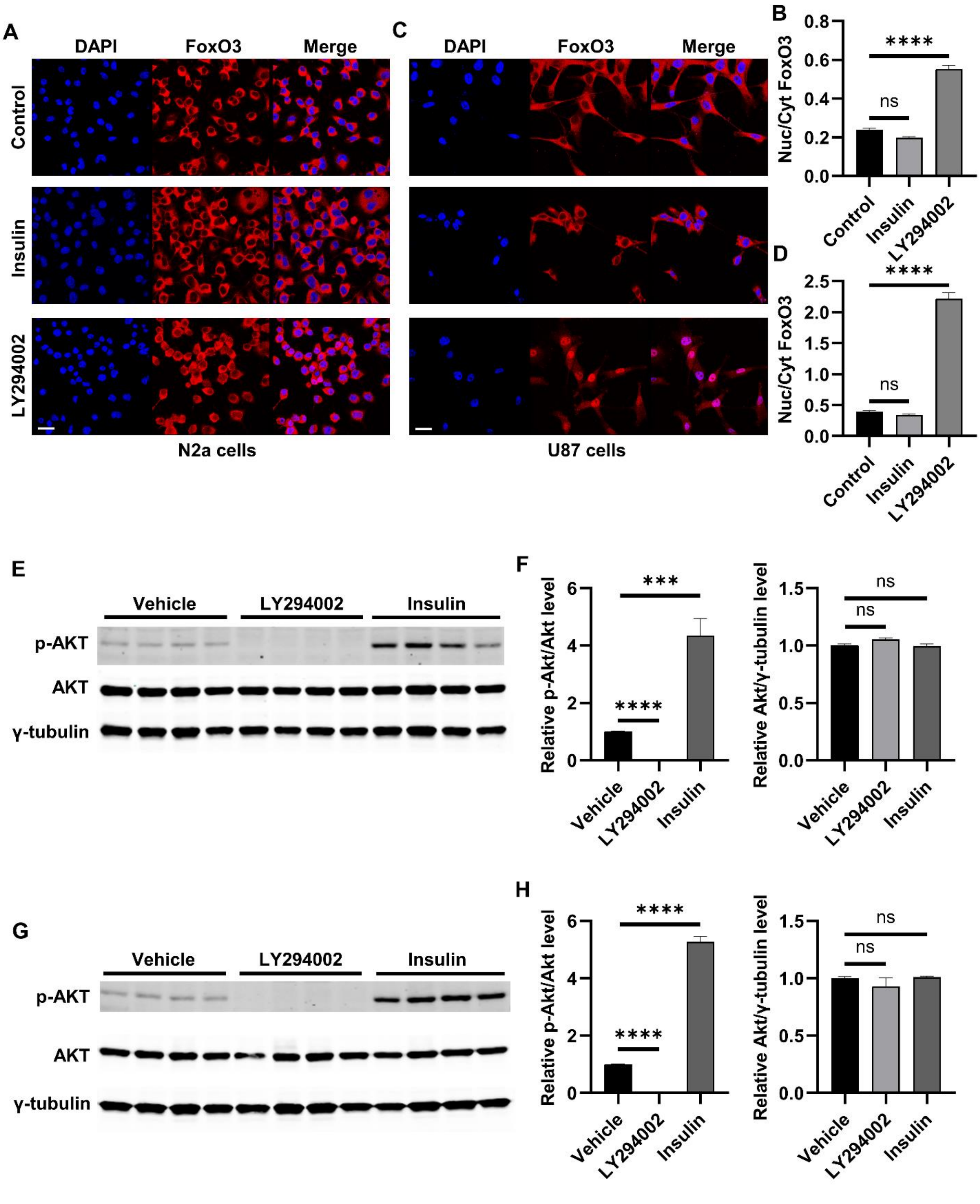

A
DAPI
FoxO3
Merge
Control
Insulin
LY294002
N2a cells
C
U87 cells
B
Nuc/Cyt FoxO3
ns
****
D
E
Vehicle
p-AKT
AKT
γ-tubulin
F
Relative p-Akt/Akt level
***
Relative Akt/γ-tubulin level
G
H

Figure S1. Cell-type specific regulation of FoxO3 and effect of LY294002 and insulin treatment. A: Representative confocal images showing FoxO3 subcellular localization in N2a cells via immunofluorescent staining after control (vehicle), insulin and LY294002 treatment. Scale bar: 25 µm. **B:** Quantification of nuclear/cytoplasmic FoxO3 ratio in **(A)**. $N_{Control}$=26; $N_{Insulin}$=28; $N_{LY294002}$=29. **C:** Representative confocal images showing FoxO3 subcellular localization in U87 cells via immunofluorescent staining after control (vehicle), insulin and LY294002 treatment. Scale bar: 25 µm. **D:** Quantification of nuclear/cytoplasmic FoxO3 ratio in **(C)**. $N_{Control}$=26; $N_{Insulin}$=23; $N_{LY294002}$=25. **E:** Representative Western blots showing phospho-AKT and total AKT protein levels in primary neurons after control (vehicle), insulin and LY294002 treatment. γ-tubulin was used as the internal control. **F:** Quantification showing the level of AKT phosphorylation (left panel) and total AKT expression (right panel) in **(E)**. N=4. **G:** Representative Western blots showing phospho-AKT and total AKT protein levels in primary astrocytes after control (vehicle), insulin and LY294002 treatment. γ-tubulin was used as the internal control. **H:** Quantification showing the level of AKT phosphorylation (left panel) and total AKT expression (right panel) in **(G)**. N=4. Data are presented as mean ± SEM. Significance determined by one-way ANOVA with Tukey's multiple comparisons test. ns, not significant, ***p<0.001, ****p<0.0001.

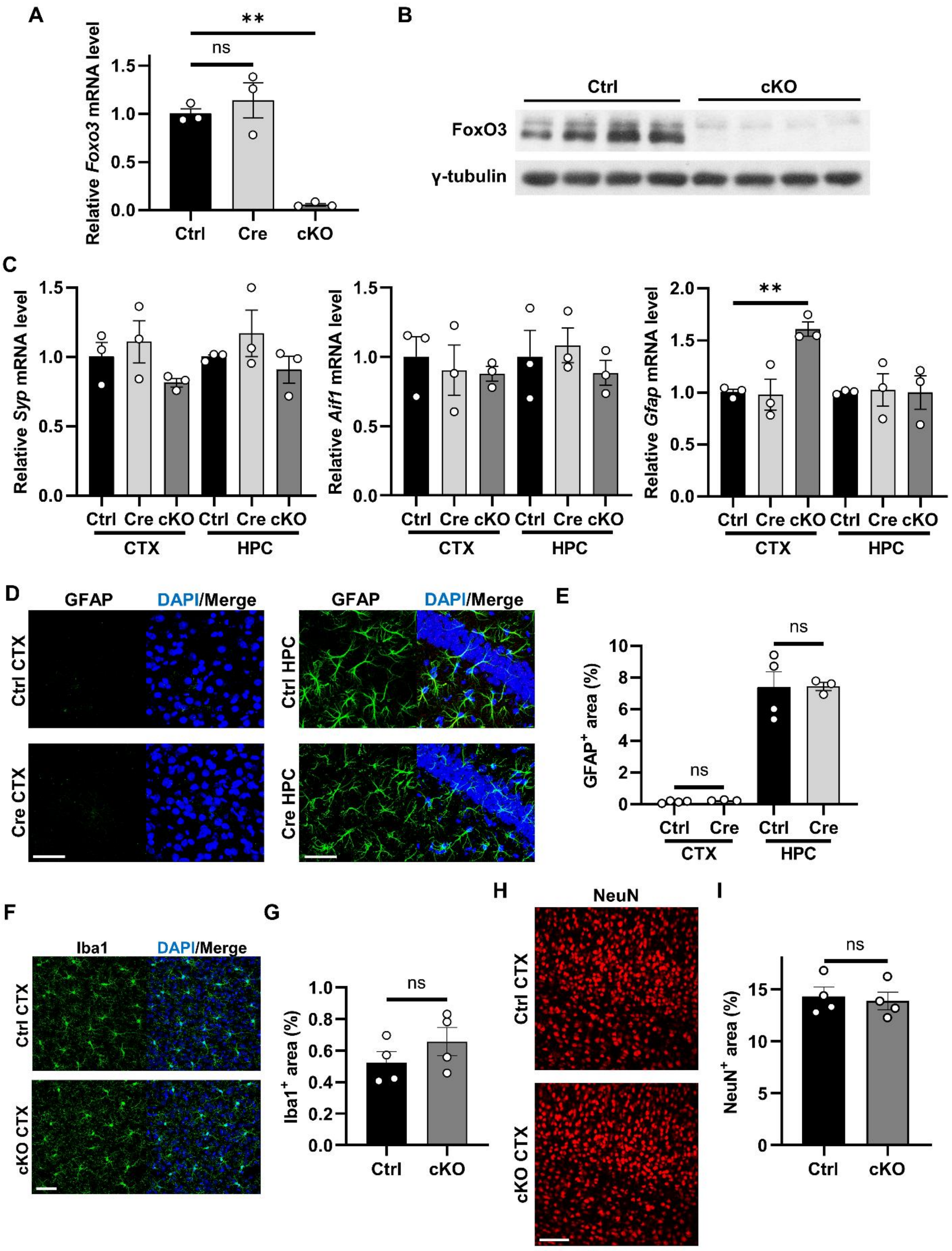

A
Relative Foxo3 mRNA level
**
ns
1.5
1.0
0.5
0.0
Ctrl
Cre
cKO
B
Ctrl
cKO
FoxO3
γ-tubulin
C
Relative Syp mRNA level
Relative Aif1 mRNA level
Relative Gfap mRNA level
**
Ctrl Cre cKO Ctrl Cre cKO
CTX
HPC
D
GFAP
DAPI/Merge
Ctrl CTX
Cre CTX
Ctrl HPC
Cre HPC
E
GFAP+ area (%)
ns
F
Iba1
DAPI/Merge
Ctrl CTX
cKO CTX
G
Iba1+ area (%)
ns
H
NeuN
Ctrl CTX
cKO CTX
I
NeuN+ area (%)
ns

Figure S2. Expression analysis and cell marker staining of Ctrl, Cre and cKO mice. A: qPCR analysis of the mRNA level of *Foxo3* in the cortex from Ctrl, Cre and cKO mice at 3 months of age. N=3. **B:** Western blot showing the protein level of FoxO3 in the forebrain samples from FoxO3 cKO and Ctrl mice at 3 months of age. N=4. **C:** qPCR analysis of the mRNA levels of *Syn* (synaptophysin), *Aif1* (allograft inflammatory factor 1, also known as Iba1) and *Gfap* (glial fibrillary acidic protein) in both CTX and HPC of Ctrl, Cre and cKO mice at 3 months of age. N=3. **D:** Representative confocal images showing cortical and hippocampal astrocytes via immunofluorescent staining of GFAP in Ctrl and Cre brain sections at 3 months of age. Scale bar: 50 µm. **E:** Quantification of the GFAP positive percentage area in **(D)**. N_{Ctrl}=4; N_{Cre}=3. **F:** Representative confocal images showing cortical microglia via immunofluorescent staining of Iba1 in cKO and Ctrl brain sections at 3 months of age. Scale bar: 50 µm. **G:** Quantification of the Iba1 positive percentage area in **(F)**. N=4. **H:** Representative confocal images showing cortical neurons via immunofluorescent staining of NeuN in cKO and Ctrl brain sections at 3 months of age. Scale bar: 100 µm. **I:** Quantification of the NeuN positive percentage area in **(H)**. N=4. Male mice were used in all groups. Data are presented as mean ± SEM. Significance determined by Student's t test or one-way ANOVA with Tukey's multiple comparisons test. ns, not significant, **p<0.01.

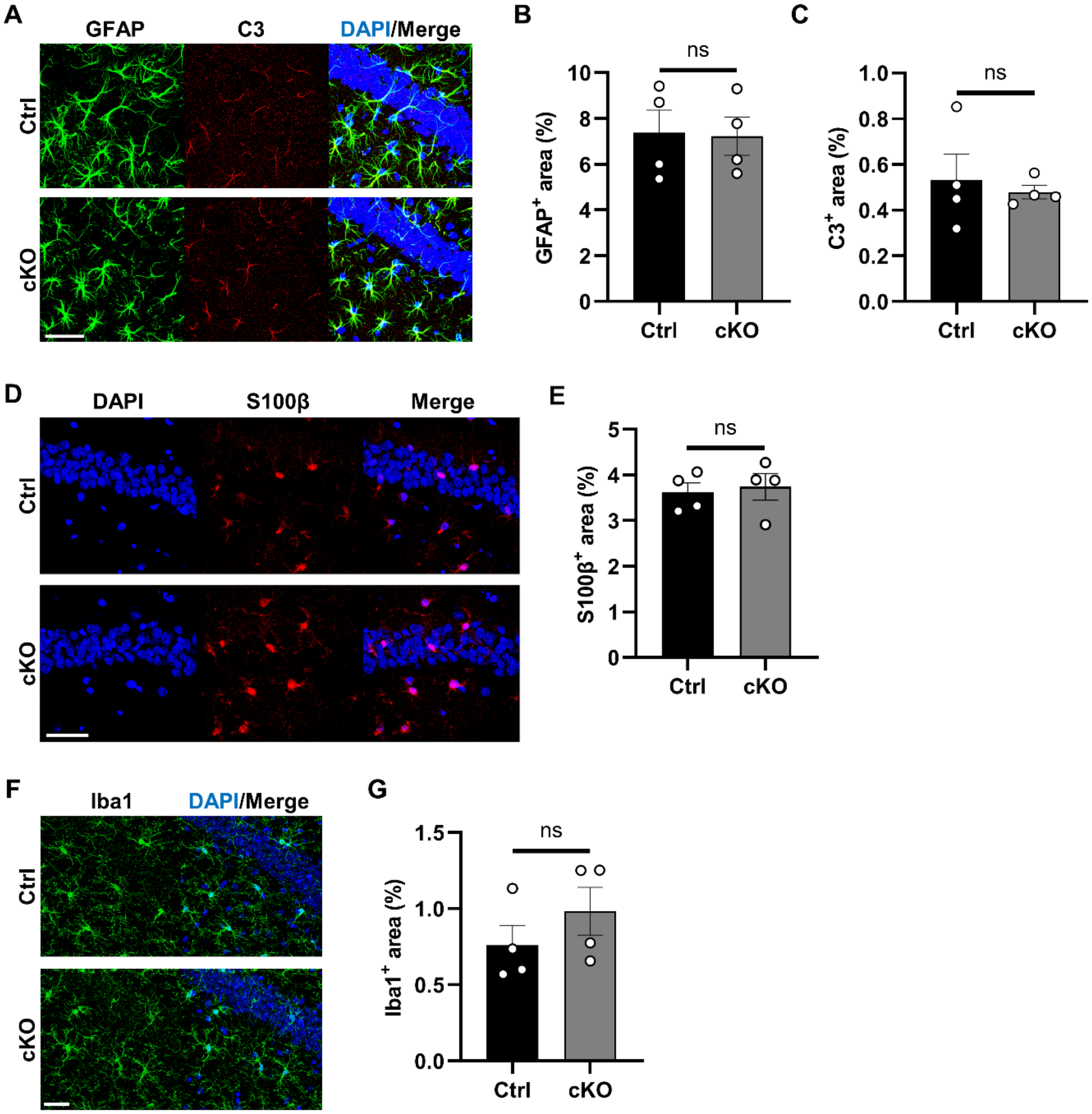

A
GFAP
C3
DAPI/Merge
Ctrl
cKO
B
ns
GFAP+ area (%)
10
8
6
4
2
0
Ctrl
cKO
C
ns
C3+ area (%)
1.0
0.8
0.6
0.4
0.2
0.0
Ctrl
cKO
D
DAPI
S100β
Merge
Ctrl
cKO
E
ns
S100β+ area (%)
5
4
3
2
1
0
Ctrl
cKO
F
Iba1
DAPI/Merge
Ctrl
cKO
G
ns
Iba1+ area (%)
1.5
1.0
0.5
0.0
Ctrl
cKO

Figure S3. Immunostaining of astrocytes and microglia in the hippocampus. A: Representative confocal images showing hippocampal astrocytes via immunofluorescent co-staining of GFAP and C3 in cKO and Ctrl brain sections at 3 months of age. Scale bar: 50 μm. **B:** Quantification of the GFAP positive percentage area in **(A)**. N=4. **C:** Quantification of the C3 positive percentage area in **(A)**. N=4. **D:** Representative confocal images showing hippocampal astrocytes via immunofluorescent staining of S100β in cKO and Ctrl brain sections at 3 months of age. Scale bar: 50 μm. **E:** Quantification of the S100β positive percentage area in **(D)**. N=4. **F:** Representative confocal images showing hippocampal microglia via immunofluorescent staining of Iba1 in cKO and Ctrl brain sections at 3 months of age. Scale bar: 50 μm. **G:** Quantification of the Iba1 positive percentage area in **(F)**. N=4. Male mice were used in all groups. Data are presented as mean ± SEM. Significance determined by Student's t test. ns, not significant.

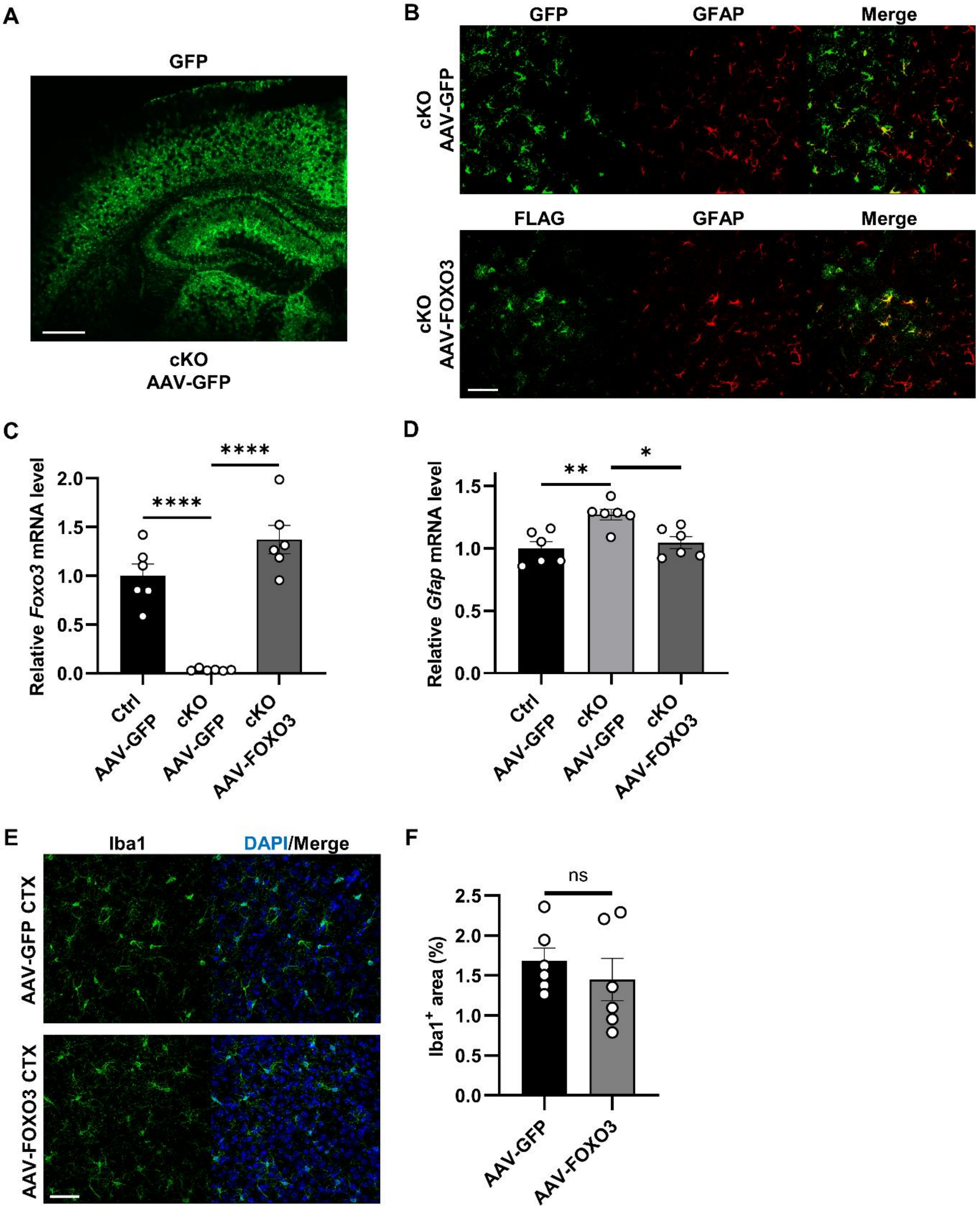
A
GFP
cKO
AAV-GFP
B
GFP
GFAP
Merge
cKO
AAV-GFP
FLAG
GFAP
Merge
cKO
AAV-FOXO3
C
Relative Foxo3 mRNA level
2.0
1.5
1.0
0.5
0.0
****
****
Ctrl
AAV-GFP
cKO
AAV-GFP
cKO
AAV-FOXO3
D
Relative Gfap mRNA level
1.5
1.0
0.5
0.0
**
*
Ctrl
AAV-GFP
cKO
AAV-GFP
cKO
AAV-FOXO3
E
Iba1
DAPI/Merge
AAV-GFP CTX
AAV-FOXO3 CTX
F
ns
Iba1+ area (%)
2.5
2.0
1.5
1.0
0.5
0.0
AAV-GFP
AAV-FOXO3

Figure S4. Characterization of astrocytic expression of AAV-GFP and AAV-FOXO3. A: A representative EVOS image showing GFP expression in the AAV-GFP injected *Foxo3* cKO mice. Scale bar: 0.5 mm. **B:** Representative confocal images showing colocalization of GFP and GFAP labeled astrocytes via immunofluorescent staining of GFAP in brain sections of *Foxo3* cKO mice with AAV-GFP injection (upper panel) and colocalization of FOXO3 and GFAP labeled astrocytes via co-staining of FLAG and GFAP in brain sections of *Foxo3* cKO mice with AAV-FOXO3 injection at 3 months of age (lower panel). Scale bar: 100 μm. **C and D:** qPCR analysis of the mRNA levels of *Foxo3* **(C)** and *Gfap* **(D)** in the cortex of Ctrl mice with AAV-GFP injection, *Foxo3* cKO mice with AAV-GFP injection and *Foxo3* cKO mice with AAV-FOXO3 injection. N=6. **E:** Representative confocal images showing cortical microglia via immunofluorescent staining of Iba1 in brain sections from 3-month-old *Foxo3* cKO mice with AAV-FOXO3 or AAV-GFP injections. Scale bar: 50 μm. **F:** Quantification of the Iba1 positive percentage area in **(E)**. N=6. Male mice were used in all groups. Data are presented as mean ± SEM. Significance determined by Student's t test or one-way ANOVA with Tukey's multiple comparisons test. ns, not significant, *$p<0.05$, **$p<0.01$, ****$p<0.0001$.

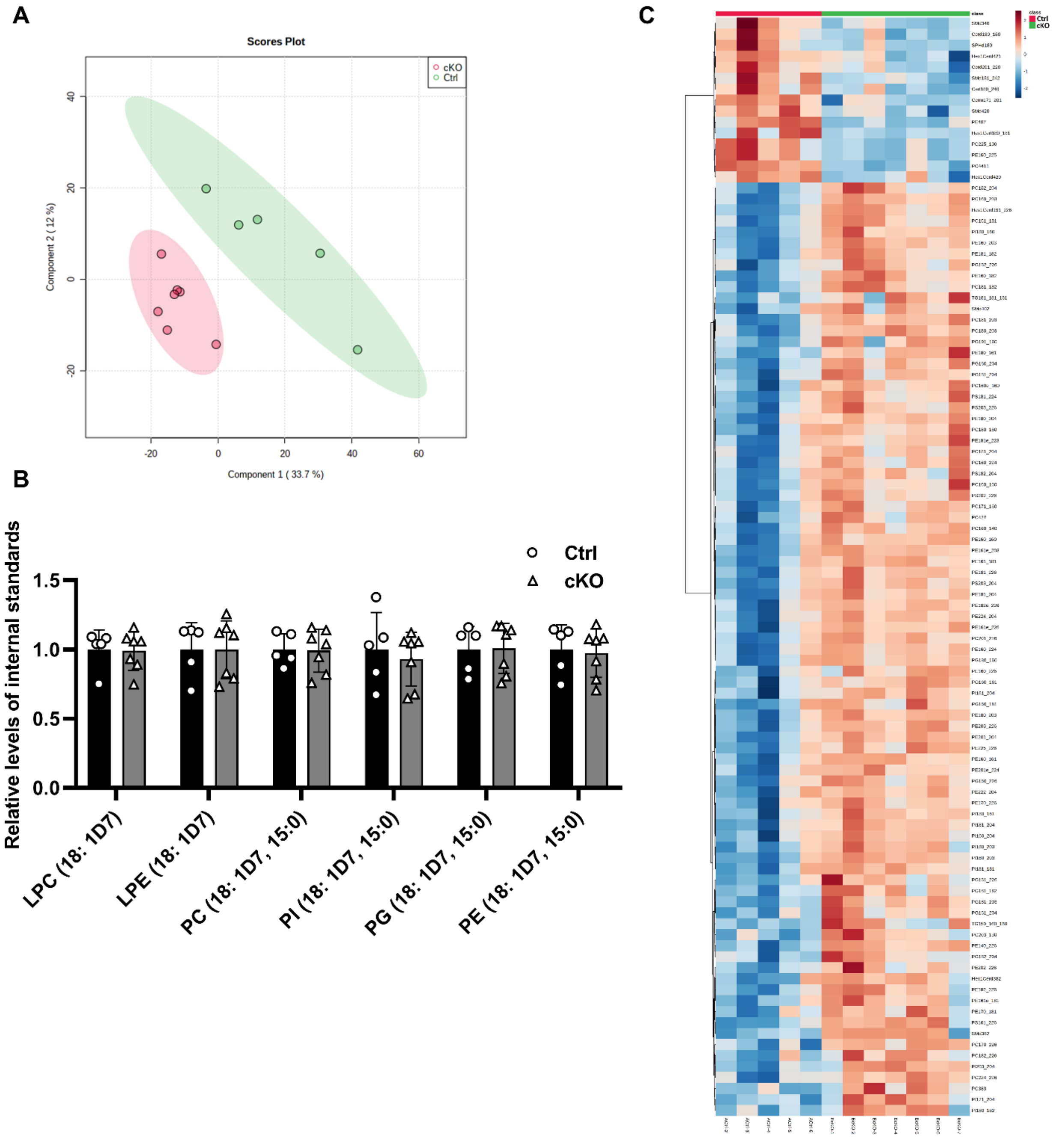

Figure S5. General characterization of mass-spec lipodomics. A: Partial least squares discriminant analysis (PLS-DA) showing the separate clustering of *Foxo3* cKO and Ctrl groups. **B:** Relative detected levels of internal standards spiked in each sample during preparation. **C:** Heatmap showing the expression of the top 100 lipid molecules with most significant p-values in each sample.

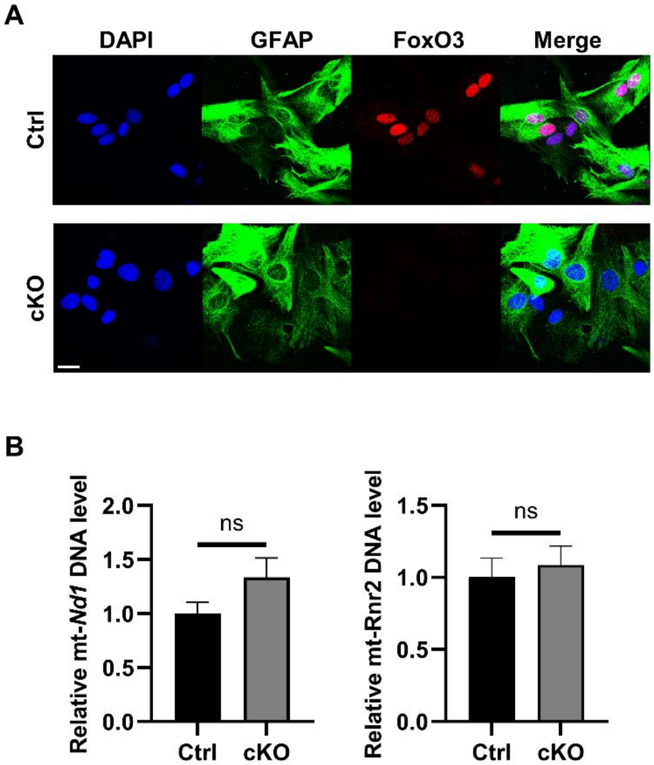

Figure S6. Characterization of primary astrocyte cultures. A: Representative confocal images showing GFAP and FoxO3 immunostaining in *Foxo3* cKO and Ctrl primary astrocytes. Scale bar: 25 µm. **B:** qPCR analysis of the relative DNA levels of mitochondrial genes *Nd1 and Rnr2* in *Foxo3* cKO and Ctrl primary astrocytes. N=6. Data are presented as mean ± SEM. Significance determined by Student's t test. ns, not significant.

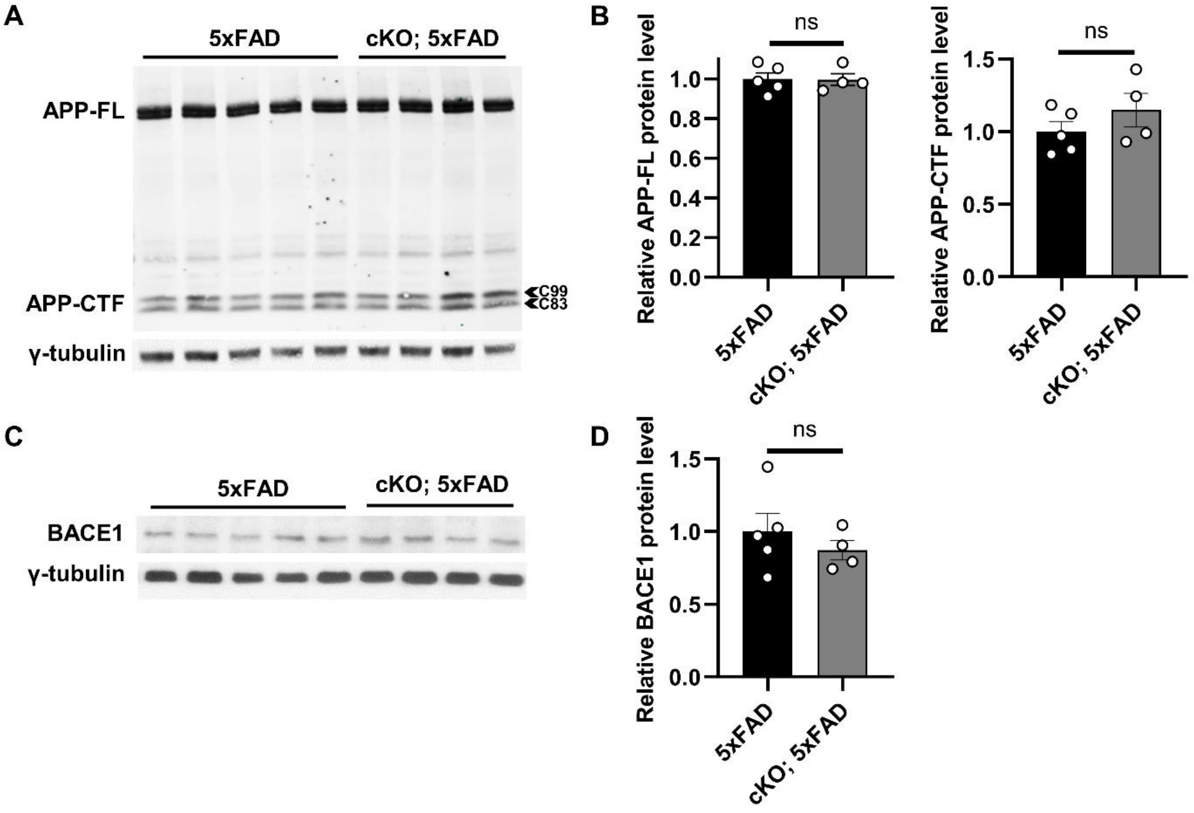

Figure S7. Expression and cleavage of APP were not affected by FoxO3 deficiency. A: Western blot of the protein levels of full-length APP (APP-FL) and APP C-terminal fragments (APP-CTF) in the forebrain lysates from *Foxo3* cKO; 5xFAD and 5xFAD mice at the age of 5 months. γ-tubulin was used as the internal control. **B:** Quantification of the levels of APP-FL and APP-CTF against γ-tubulin. N_{5xFAD}=5; $N_{cKO;\ 5xFAD}$=4. **C:** Western blot of the protein level of BACE1 in the forebrain lysates from *Foxo3* cKO; 5xFAD and 5xFAD mice at the age of 5 months. γ-tubulin was used as the internal control. **D:** Quantification of the level of BACE1 against γ-tubulin. N_{5xFAD}=5; $N_{cKO;\ 5xFAD}$=4. Male mice were used in both groups. Data are presented as mean ± SEM. Significance determined by Student's t test. ns, not significant.

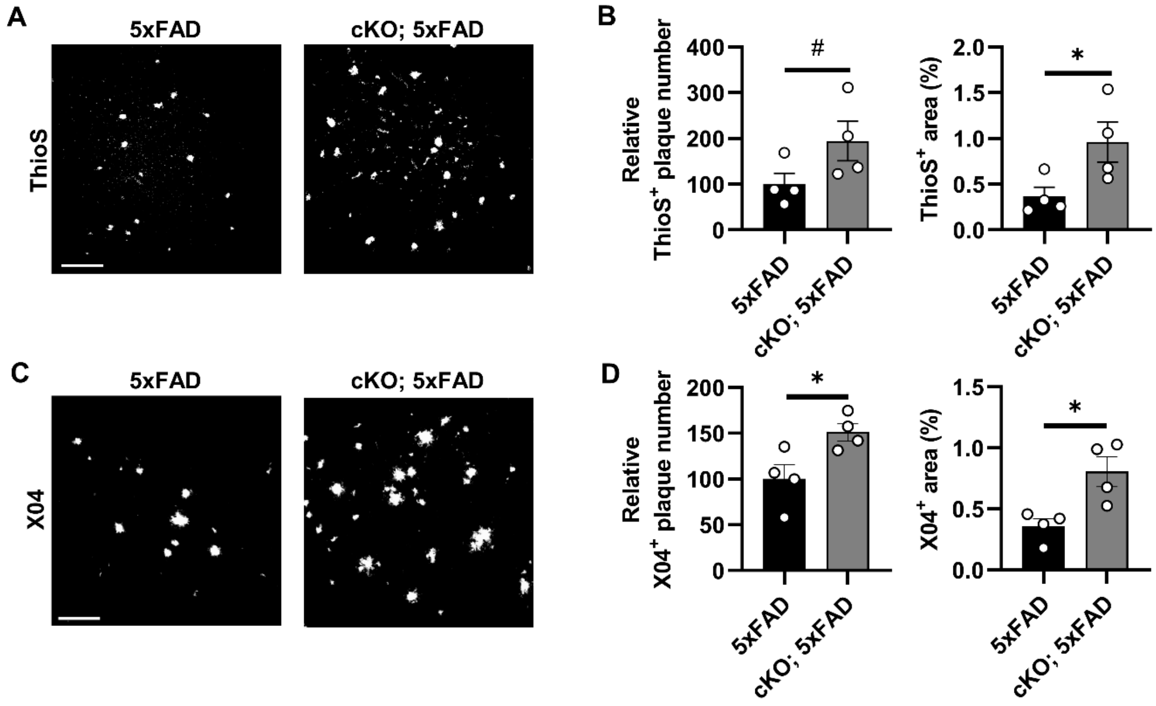

Figure S8. Analysis of Aβ pathology in 5xFAD mice with FoxO3 deficiency. A: Representative confocal images showing amyloid plaques in the cortex by ThioS staining in *Foxo3* cKO; 5xFAD and 5xFAD male mice at 5 months of age. Scale bar: 100 μm. **B:** Quantification of ThioS positive plaque number and percentage area in the cortex. N=4. **C:** Representative confocal images showing amyloid plaques in the cortex by X04 staining in *Foxo3* cKO; 5xFAD and 5xFAD female mice at 3 months of age. Scale bar: 100 μm. **D:** Quantification of X04 positive plaque number and percentage area in the cortex. N=4. Data are presented as mean ± SEM. Significance determined by Student's t test. ns, not significant, #p<0.10, *p<0.05.

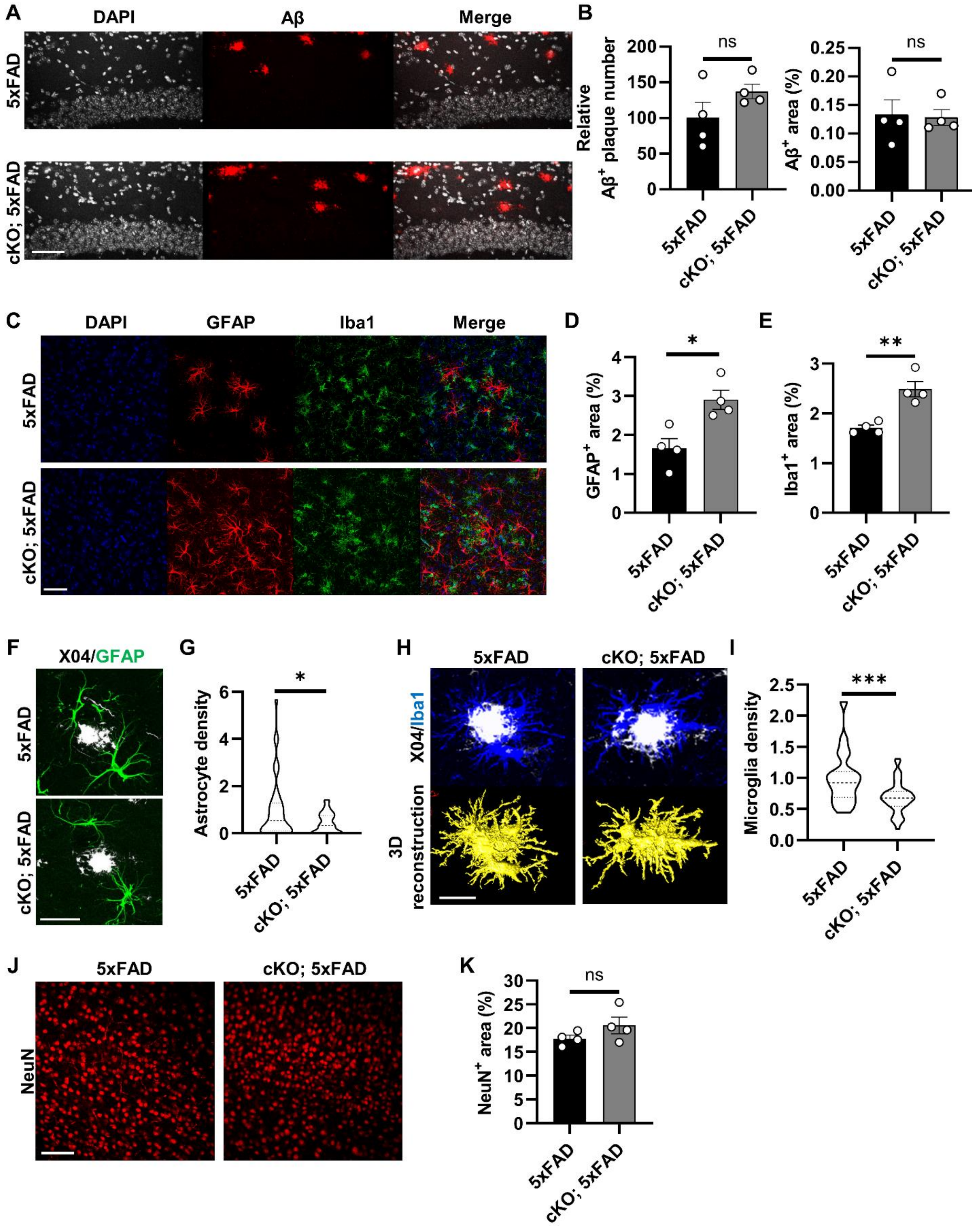
A
DAPI
Aβ
Merge
5xFAD
cKO; 5xFAD
B
Relative Aβ+ plaque number
Aβ+ area (%)
ns
ns
C
DAPI
GFAP
Iba1
Merge
D
GFAP+ area (%)
*
E
Iba1+ area (%)
**
F
X04/GFAP
G
Astrocyte density
*
H
X04/Iba1
3D reconstruction
I
Microglia density
***
J
NeuN
K
NeuN+ area (%)
ns

Figure S9. Analysis of Aβ and associated pathology in 5xFAD mice with FoxO3 deficiency. A: Representative confocal images showing amyloid plaques in the hippocampus by Aβ immunostaining in *Foxo3* cKO; 5xFAD and 5xFAD mice. Scale bar: 100 µm. **B:** Quantification of Aβ positive plaque number and percentage area in the hippocampus. N=4. **C:** Representative confocal images showing reactive astrocytes and microglia in the cortex by GFAP and Iba1 co-staining in *Foxo3* cKO; 5xFAD and 5xFAD mice. Scale bar: 100 µm. **D:** Quantification of GFAP positive percentage area in **(C)**. N=4. **E:** Quantification of Iba1 positive percentage area in **(C)**. N=4. **F:** Representative confocal images showing amyloid plaques and plaque-associated reactive astrocytes via X04 and GFAP co-staining in *Foxo3* cKO; 5xFAD and 5xFAD mice. Scale bar: 30 µm. **G:** Quantification of astrocyte density defined by the ratio of the total volume of astrocytes within 50 µm of their associated plaque over the volume of the plaque in **(F)** using IMARIS software. N_{5xFAD}=40; $N_{cKO;\ 5xFAD}$=39. **H:** Representative confocal images showing amyloid plaques and plaque-associated microglia via X04 and Iba1 co-staining in *Foxo3* cKO; 5xFAD and 5xFAD mice (upper panel) and the 3D reconstruction of plaque-associated microglia in Imaris (yellow, lower panel). Scale bar: 20 µm. **I:** Quantification of microglia density defined by the ratio of the total volume of plaque-associated microglia over the volume of the plaque in **(H)** using IMARIS software. N_{5xFAD}=41; $N_{cKO;\ 5xFAD}$=40. **J:** Representative confocal images showing cortical neurons via immunofluorescent staining in *Foxo3* cKO; 5xFAD and 5xFAD mice. Scale bar: 100 µm. **K:** Quantification of the NeuN positive percentage area in **(J)**. N=4. Male mice at 3.5 months were used in all experiments. Data are presented as mean ± SEM. Significance determined by Student's t test. ns, not significant, *p<0.05, **p<0.01, ***p<0.001.

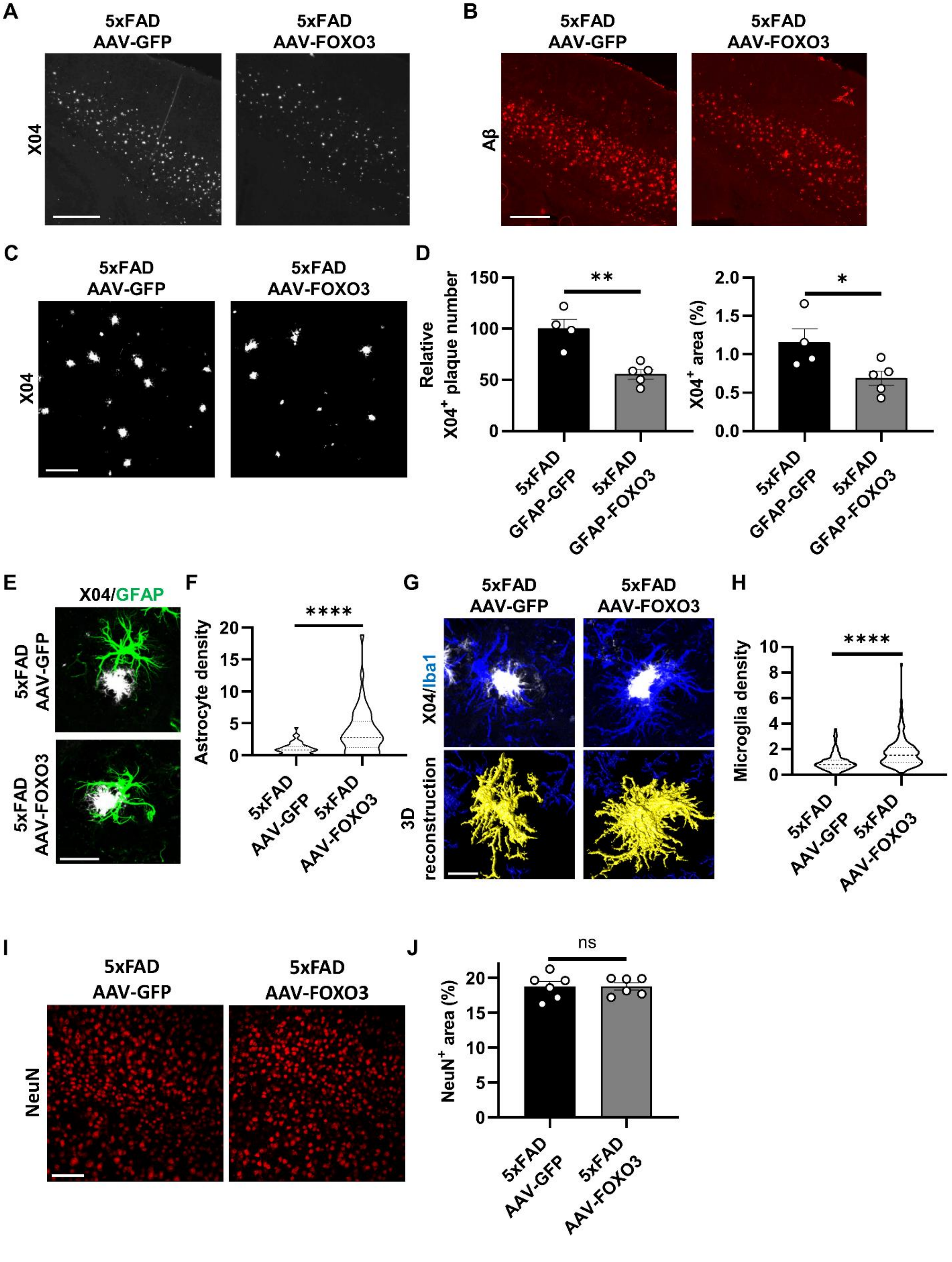
A
5xFAD AAV-GFP
5xFAD AAV-FOXO3
X04
B
5xFAD AAV-GFP
5xFAD AAV-FOXO3
Aβ
C
5xFAD AAV-GFP
5xFAD AAV-FOXO3
X04
D
**
Relative X04+ plaque number
150
100
50
0
5xFAD GFAP-GFP
5xFAD GFAP-FOXO3
*
X04+ area (%)
2.0
1.5
1.0
0.5
0.0
5xFAD GFAP-GFP
5xFAD GFAP-FOXO3
E
X04/GFAP
5xFAD AAV-GFP
5xFAD AAV-FOXO3
F
****
Astrocyte density
20
15
10
5
0
5xFAD AAV-GFP
5xFAD AAV-FOXO3
G
5xFAD AAV-GFP
5xFAD AAV-FOXO3
X04/Iba1
3D reconstruction
H
****
Microglia density
10
8
6
4
2
0
5xFAD AAV-GFP
5xFAD AAV-FOXO3
I
5xFAD AAV-GFP
5xFAD AAV-FOXO3
NeuN
J
ns
NeuN+ area (%)
20
15
10
5
0
5xFAD AAV-GFP
5xFAD AAV-FOXO3

Figure S10. Analysis of Aβ and associated pathology in 5xFAD mice with astrocytic FoxO3 overexpression. A: Representative EVOS images showing amyloid plaques in the cortex by X04 staining of 5-month-old female 5xFAD mice with AAV-GFP or AAV-FOXO3 injections. Scale bar: 0.5 mm. **B:** Representative EVOS images showing amyloid plaques in the cortex by Aβ staining in 5-month-old female 5xFAD mice with AAV-GFP or AAV-FOXO3 injections. Scale bar: 0.5 mm. **C:** Representative confocal images showing amyloid plaques in the cortex by X04 staining of 5-month-old male 5xFAD mice with AAV-GFP or AAV-FOXO3 injections. Scale bar: 100 μm. **D:** Quantification of X04 positive plaque number and percentage area in the cortex. $N_{AAV\text{-}GFP}$=4; $N_{AAV\text{-}FOXO3}$=5. **E:** Representative confocal images showing amyloid plaques and plaque-associated reactive astrocytes via X04 and GFAP co-staining of 5-month-old female 5xFAD mice with AAV-GFP or AAV-FOXO3 injections. Scale bar: 30 μm. **F:** Quantification of astrocyte density defined by the ratio of the total volume of astrocytes within 50 μm of their associated plaque over the volume of the plaque in **(E)** using IMARIS software. $N_{AAV\text{-}GFP}$=45; $N_{AAV\text{-}FOXO3}$=48. **G:** Representative confocal images showing amyloid plaques and plaque-associated microglia via X04 and Iba1 co-staining of 5-month-old female 5xFAD mice with AAV-GFP or AAV-FOXO3 injections (upper panel) and the 3D reconstruction of plaque-associated microglia in IMARIS (yellow, lower panel). Scale bar: 20 μm. **H:** Quantification of microglia density defined by the ratio of the total volume of plaque-associated microglia over the volume of the plaque in **(G)** using IMARIS software. $N_{AAV\text{-}GFP}$=202; $N_{AAV\text{-}FOXO3}$=150. **I:** Representative confocal images showing cortical neurons via immunofluorescent staining in 5-month-old female 5xFAD mice with AAV-GFP or AAV-FOXO3 injections. Scale bar: 100 μm. **J:** Quantification of the NeuN positive percentage area in **(I)**. N=6. Data are presented as mean ± SEM. Significance determined by Student's t test. ns, not significant, *p<0.05, **p<0.01, ****p<0.0001.
